# New insights on interpersonal violence in the Late Pleistocene based on the Nile valley cemetery of Jebel Sahaba

**DOI:** 10.1038/s41598-021-89386-y

**Published:** 2021-05-27

**Authors:** Isabelle Crevecoeur, Marie-Hélène Dias-Meirinho, Antoine Zazzo, Daniel Antoine, François Bon

**Affiliations:** 1 UMR 5199-PACEA, CNRS, Université de Bordeaux, B8, Allée Geoffroy Saint-Hilaire, CS 50023, 33615 Pessac Cedex, France; 2 UMR 5608-TRACES, Université de Toulouse Jean Jaurès, Maison de La Recherche, 5 Allées Antonio Machado, 31058 Toulouse Cedex 9, France; 3 UMR 7209-AASPE, CNRS, MNHN, CP 56 - 43 Rue Buffon, 75005 Paris, France; 4 Department of Egypt and Sudan, The British Museum, Great Russell Street, London, WC1B 3DG UK

**Keywords:** Archaeology, Biological anthropology

## Abstract

The remains of 61 individuals buried in the cemetery of Jebel Sahaba (site 117) offer unique and substantial evidence to the emergence of violence in the Nile Valley at the end of the Late Pleistocene. Excavated and assessed in the 1960s, some of the original findings and interpretations are disputed. A full reanalysis of the timing, nature and extent of the violence was conducted through the microscopic characterization of the nature of each osseous lesion, and the reassessment of the archaeological data. Over 100 previously undocumented healed and unhealed lesions were identified on both new and/or previously identified victims, including several embedded lithic artefacts. Most trauma appears to be the result of projectile weapons and new analyses confirm for the first time the repetitive nature of the interpersonal acts of violence. Indeed, a quarter of the skeletons with lesions exhibit both healed and unhealed trauma. We dismiss the hypothesis that Jebel Sahaba reflects a single warfare event, with the new data supporting sporadic and recurrent episodes of inter-personal violence, probably triggered by major climatic and environmental changes. At least 13.4 ka old, Jebel Sahaba is one of the earliest sites displaying interpersonal violence in the world.

## Introduction

The end of the Late Pleistocene and the beginning of the Holocene were marked by major climatic changes whose impact on human populations is still poorly understood (^1–3^; cf. Supplementary Text S1). In the Nile Valley, climatic conditions are depicted as hyper-arid during the second half of the Late Pleistocene^4^. Around 15–14 ka, the sudden overflow of Lake Victoria into the White Nile establishes the present Nile-flow regime, causing regular and severe flooding all the way down to Egypt^5,6^. Only after the Younger Dryas (~ 12.9–11.7 ka), do the monsoon conditions of the African Humid Period start to stabilize^3^. There is little evidence for human occupations at the end of the Late Pleistocene (~ 18–11.7 ka) in the Nile Valley, with sites restricted to the floodplain of Upper Egypt and Nubia^7–9^ (cf. Fig. S1). Of these, few have yielded complete human remains. These include Jebel Sahaba (Site 117), Tushka (Site 8905), Wadi Kubbaniya, and site 6-B-36 from Wadi Halfa^10–12^.

Culturally, different lithic industries have been identified with sites associated to the end of the Late Pleistocene e.g.^13–17^. These occur in restricted geographical areas along the Nile, mainly in Upper Egypt. They do not seem to be related to specific activities and are defined by characteristic sets of lithic tools and/or technology that appear to be associated with distinct small hunting-fishing-gathering groups^15–17^. Each of these lithic groups is believed to represent a cultural tradition that reflects group identity^15^ (cf. Supplementary Text S1 and Fig. S1). The occurrence of large graveyards at the end of the Late Pleistocene reinforces the idea of strong social units within these residential groups^18^.

Set in a context of possible environmental pressures and geographical constraints, the identification of traces of interpersonal violence on the individuals buried in Jebel Sahaba have attracted much attention^18,19^. Evidence of conflicts is not uncommon in the Nile valley. The oldest documented case (~ 20 ka) appears to be from Wadi Kubbaniya, where the remains of a partial skeleton belonging to a young adult male provides early evidence of interpersonal violence (^12,20^). Embedded lithic and healed fractures have also been documented on some individuals buried in the Wadi Halfa cemetery, associated with Qadan lithic industry (site 6-B-36;^10,21^). However, the most emblematic and widely cited example of early widespread violence is the cemetery of Jebel Sahaba. Dated between 13,400 and 18,600 cal BP (10,032 ± 46 BP, UBA-20131, to 13,740 ± 600 BP, Pta-116), the Jebel Sahaba cemetery is the earliest funerary complex from the Nile Valley (cf. Supplementary Text S2 and Table S1). Early analyses of the skeletons by Anderson^19^ and Butler^22^ revealed evidence of interpersonal violence on the bones of at least half of the Jebel Sahaba individuals. In addition, abundant lithic artefacts from the Qadan industry were discovered within the physical space of the bodies, where the soft tissues would have once been, or directly embedded in the bones^23^. Given their position, these lithic artefacts cannot be considered to be grave goods, nor can the Jebel Sahaba individuals be referred to as belonging to a Qadan population, particularly as other cultural entities are present in Lower Nubia during the same period^17^.

Since its discovery and original publication by Wendorf^11^, the cemetery has been cited as a key example for the emergence of violence and organized warfare triggered by territorial disputes^24–28^. Many elements of the original findings, particularly the timing, nature and extent of the violence, but also the lithic association, have since been challenged e.g.^29–32^. However, no holistic study of the traces of violence left on the human remains has been undertaken to reassess the site and provide an updated perspective on violence and human behavior at the end of the Late Pleistocene^26^. Here we address several unanswered questions that benefit from a full reanalysis of the collection using the latest anthropological and forensic methods. Indeed, it remains unclear whether the cemetery was the result of a single event, of sporadic or repetitive episodes of inter-personal violence, or was used as a place for the burial of specific individuals. Some cutmarks appear to be the result of projectile penetration while others are thought to have been caused by deliberate cuts as part of specific mortuary treatments. Finally, a reassessment of the lithic assemblage would also further our understanding of the site.

## Results

**Figure 1:**
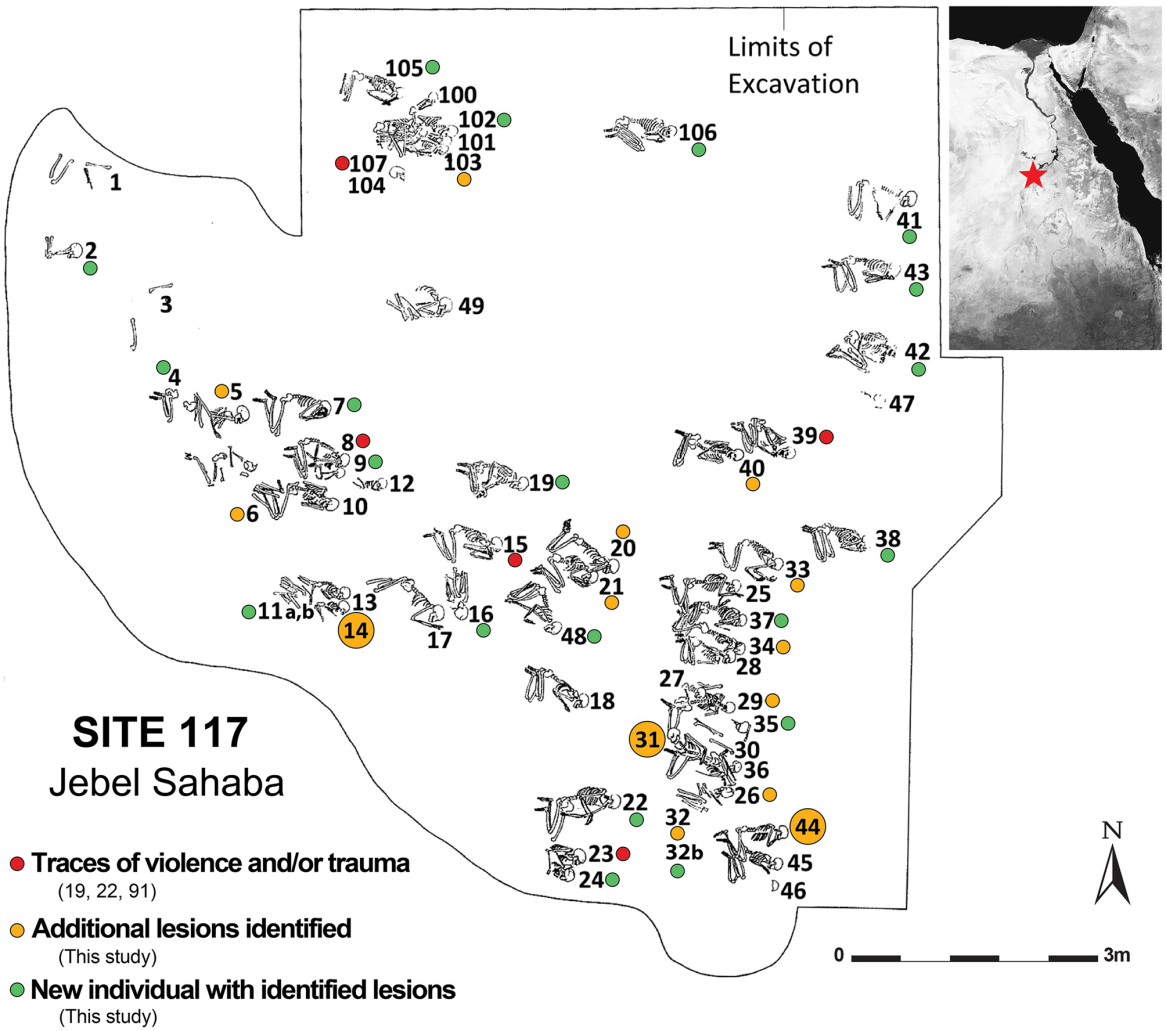
Location of the Jebel Sahaba cemetery, Site 117, in the Nile Valley and map of the excavated area and burials (map following^23^, Image Courtesy British Museum Wendorf Archive, modified with Adobe Illustrator CS6, https://www.adobe.com/products/illustrator.html). Red dots: individuals exhibiting signs of violence and/or traumatic lesions^19,20,91^; orange dots: newly identified lesions in the latter individuals; green dots: individuals newly identified as showing signs of violence and/or traumatic lesions; large dots: individuals discussed in detail in the text. Satellite image: Google Maps, 2020. 21° 58′ 12.0" N 31° 22′ 12.0" E, elevation 21.9 M. [online] Available through: <https://www.google.com/maps/place/>.

The individuals examined and the occurrence of healed and unhealed lesions and traumas are listed in the Supplementary Table S2. A systematic macroscopic and microscopic analysis confirmed most of the lesions originally described by Anderson^19^ and Butler^22^, and allowed the identification of a substantial number of additional traumas and lesions in new and previously identified individuals (identified by green and orange dots in Fig. 1).

### Reassessment of the evidence of interpersonal violence

A further 106 previously unidentified lesions were observed, including 52 that can now be interpreted as Projectile Impact Marks (PIMs). They reveal that a further twenty-one individuals had clear signs of interpersonal trauma in addition to the twenty described by Wendorf^23^ and Anderson^19^. Of the sixty-one individuals studied, forty-one (67.2%) exhibit at least one type of healed or unhealed lesion (Table 1). This includes three-quarters of the adults (74.4%, n = 32), and half of the non-adults affected (50%; n = 9). Our analyses also show that out of these sixty-one individual, 26.2% (n = 16) had signs of perimortem traumas (*i.e.* unhealed traumas and/or PIMs), and 62.3% (n = 38) displayed healed and/or unhealed traumas.

**Table 1:** Number of individuals exhibiting at least one type of lesion grouped by age-at-death or sexual diagnosis.

	Total (n = 61)		Mature (n = 43)		Immature (n = 18)												Female (n = 19)		Male (n = 20)		Und. (n = 6)	
					Total		[0– < 1] (n = 2)		[1–4] (n = 5)		[5–9] (n = 6)		[10–14] (n = 3)		[15–19] (n = 2)							
	n	%	n	%	n	%	n	%	n	%	n	%	n	%	n	%	n	%	n	%	n	%
**No lesion**	20	32.8	11	25.6	9	50.0	2	100.0	3	60.0	4	66.7	0	0.0	0	0.0	5	26.3	5	25.0	1	16.7
**Lesions**	**41**	**67.2**	32	**74.4**	9	**50.0**	0	**0.0**	2	**40.0**	2	**33.3**	3	**100.0**	2	**100.0**	14	**73.7**	15	**75.0**	5	**83.3**
Healed lesions	38	92.7	32	100.0	5	55.6	0	0.0	0	0.0	1	50.0	2	66.7	2	100.0	14	100.0	15	100.0	5	100.0
Unhealed lesions	19	46.3	15	46.9	5	55.6	0	0.0	2	100.0	1	50.0	1	33.3	1	50.0	8	57.1	8	53.3	0	0.0
H&U lesions	16	39.0	15	46.9	1	11.1	0	0.0	0	0.0	0	0.0	0	0.0	1	50.0	8	57.1	8	53.3	0	0.0
**1. Traumas & PIMs**	**38**	**92.7**	30	**93.8**	8	**88.9**	0	0.0	2	**100.0**	2	**100.0**	3	**100.0**	1	**50.0**	14	**100.0**	15	**100.0**	3	**60.0**
Healed Traumas & PIMs	32	84.2	27	90.0	4	50.0	0	0.0	0	0.0	1	50.0	2	66.7	0	0.0	11	78.6	15	100.0	3	100.0
Unhealed Traumas & PIMs	16	42.1	12	40.0	5	62.5	0	0.0	2	100.0	1	50.0	1	33.3	0	0.0	7	50.0	6	40.0	0	0.0
H&U Traumas & PIMs	10	26.3	9	30.0	1	12.5	0	0.0	0	0.0	0	0.0	0	0.0	1	50.0	4	28.6	6	40.0	0	0.0
**2. Fractures**	**22**	**36.1**	21	**48.8**	1	**5.6**	0	0.0	0	**0.0**	0	**0.0**	0	**0.0**	1	**50.0**	9	**47.4**	11	**55.0**	2	**33.3**
**3. PIMs**	**25**	**61.0**	19	**59.4**	6	**66.7**	0	0.0	2	**100.0**	1	**50.0**	2	**66.7**	1	**50.0**	10	**71.4**	10	**66.7**	1	**20.0**
Healed PIMs	12	48.0	10	52.6	1	16.7	0	0.0	0	0.0	0	0.0	1	50.0	0	0.0	4	40.0	6	60.0	1	100.0
Unhealed PIMs	16	64.0	12	63.2	5	83.3	0	0.0	2	100.0	1	100.0	1	50.0	1	100.0	7	70.0	6	60.0	0	0.0
H&U PIMs	3	12.0	3	15.8	0	0.0	0	0.0	0	0.0	0	0.0	0	0.0	0	0.0	1	10.0	2	20.0	0	0.0
**4. Embedded lithic**	**11**	**26.8**	9	**28.1**	2	**22.2**	0	0.0	1	**50**	0	**0.0**	1	**50**	0	**0.0**	3	**21.4**	6	**40.0**	0	**0.0**
Healed PIMs	4	36.4	4	44.4	0	0.0	0	0.0	0	0.0	0	0.0	0	0.0	0	0.0	0	0.0	4	66.7	0	0.0
Unhealed PIMs	8	72.7	6	66.7	2	100.0	0	0.0	1	100.0	0	0.0	1	100.0	0	0.0	3	100.0	3	50.0	0	0.0
H&U PIMs	1	9.1	1	11.1	0	0.0	0	0.0	0	0.0	0	0.0	0	0.0	0	0.0	0	0.0	1	16.7	0	0.0

The percentage in the two first lines are calculated on the minimal number of individuals for each category, while the percentage in the numbered bold lines are computed based on the recorded number of individual with lesions for each category. The percentage in the underlying lines represents the proportion of individuals with healed, unhealed and healed and unhealed lesion occurrence within the numbered line category. n = number; % = percentage; PIM = Projectile Impact Mark; H&U = Healed and Unhealed. Und. = mature individuals whose sexual diagnosis is undeterminate.

Both sexes have the same percentage of healed and unhealed lesions. Among the adults with traces of injuries, 36.6% (n = 15) display signs of both healed and unhealed lesions, with males (n = 8) and females (n = 8) similarly affected. Only one non-adult, an adolescent [15–19], has both healed and unhealed lesions (Table 1). Most individuals with lesions 92.7% (n = 38) had some that were traumatic in origin, and over half of these individuals had a projectile impact (61.0%; n = 25). This percentage is similar in adults and non-adults, and between males and females. Embedded lithic fragments, among which two-third are newly identified ones (n = 13, out of 20), were recorded in the PIMs of eleven individuals (26.8%, n = 11), and with a higher proportion in males (n = 6).

The location of the lesions also reveals some patterning to the traumas or PIMs (Table 2 and Supplementary Figs. S2 & S3). First, the number of healed fractures are mainly concentrated on the upper limb and the shoulder girdle (84.8%, n = 28). Fifty percent of these upper limb fracture involve the hands, with both the proximal phalanges and the metacarpals affected, and one-third are located on the forearm. Of the latter, defensive parry fractures of the ulna are the most common (cf. Table 2 and Supplementary Fig. S2;^33^). A significant difference (P(χ^2^) > 0.05) between males and females was observed, with parry fractures of left and right sides, without favoring a side, mostly found on female individuals (88.9%, n = 8). Although not significant, hand bone fractures are more frequent in male individuals (58%, n = 7).

**Table 2:** Number and type of lesions recorded on the Jebel Sahaba individuals.

	Traumas & PIMs								Total Lesions
	Traumas		PIMs					Total	
	Fractures	Perforations/BFT	Drags	Punctures	Perforations	Total	Embedded lithic		
**Number of lesions**	33	4	40	24	6	70	20	107	139
**Number of individuals**	22	4	17	14	3	25	11	38	41
% of individuals	**36.1**	**6.6**	**27.9**	**23.0**	**4.9**	**41.0**	**18.0**	**62.3**	**67.2**
**Anatomical repartition**									
**1. Cranium (%)**	**3.0**	**100.0**	**20.0**	**25.0**	**66.7**	**25.7**	**15.0**	**21.5**	**20.9**
% Frontal	–	75.0	50.0	50.0	25.0	44.4	33.3	47.8	48.3
% Parietal	–	–	–	33.3	50.0	22.2	66.7	17.4	13.8
% Temporal	–	25.0	12.5	16.7	–	11.1	0.0	13.0	13.8
% Occipital	–	–	–	–	25.0	5.6	0.0	4.3	10.3
**2. Upper limb and shoulder girdle (%)**	**84.8**	–	**35.0**	**8.3**	–	**22.9**	**10.0**	**41.1**	**36.0**
% Shoulder girdle	7.1	–	35.7	50.0	–	37.5	50.0	18.2	20.0
% Humerus	10.7	–	35.7	–	–	31.3	50.0	18.2	18.0
% Ulna	28.6	–	14.3	–	–	12.5	–	22.7	20.0
% Radius	3.6	–	14.3	–	–	12.5	–	6.8	10.0
% Forearm	32.1	–	28.6	–	–	25.0	–	29.5	30.0
% Hand bones	50.0	–	0.0	50.0	–	6.3	–	34.1	32.0
**3. Trunk (%)**	**3.0**	–	**2.5**	**16.7**	–	**7.1**	**20.0**	**5.6**	**5.8**
**4. Lower limb and pelvic girdle (%)**	**9.1**	–	**42.5**	**50.0**	**33.3**	**44.3**	**55.0**	**31.8**	**37.4**
% Coxal	–	–	5.9	66.7	–	29.0	63.6	26.5	23.1
% Femur	–	–	94.1	25.0	–	61.3	27.3	55.9	53.8
% Tibia	–	–	–	–	–	–	–	–	3.8
% Fibula	33.3	–	–	8.3	–	3.2	9.1	5.9	5.8
% Foot bone	66.7	–	–	–	100.0	6.5	–	11.8	13.5

Percentage of each of these lesions in relation to the anatomical parts in bold numbered lines, and percentage of infliction to specific bones of these anatomical parts in underlying lines. PIM = Projectile Impact Mark; BFT = Blunt Force Trauma; % = percentage.

PIMs are most commonly observed on the lower limb and on the pelvic girdle compared to other anatomical areas (44.3%, n = 70; Table 2 and Supplementary Fig. S3). Similarly, this anatomical region has the highest frequency of puncture PIMs and embedded lithic artefacts (respectively 50.0%, n = 12; and 55.0%, n = 11). The sex of the individual does not appear to have influenced the frequency of these marks on different part of the body. Drag marks are present on both upper and lower part of the body, with lower limbs marks mostly found on the femur (94.1%, n = 16) and equally distributed across males and females, as well as the left and right sides. In the upper limbs, the clavicles and humeri exhibit the highest number of projectile marks (n = 11). The direction of the strike reveals no differences between males and females, with both displaying a similar number of projectile marks that had entered from the back or the front of the body. In both sexes, several individuals (n = 6) also exhibit marks consistent with both posterior and anterior impacts. Finally, the analysis reveals that all types of traumas were observed on the cranium. However, most of the perforations caused by blunt force traumas and/or projectile impacts are observed on the cranium of non-adults (87.5% of the perforations, n = 7).

### Individual case studies

Three cases best illustrate the complexity and range of lesions found in the Jebel Sahaba individuals regardless of their age-at-death, sex or burial.

**Figure 2:**
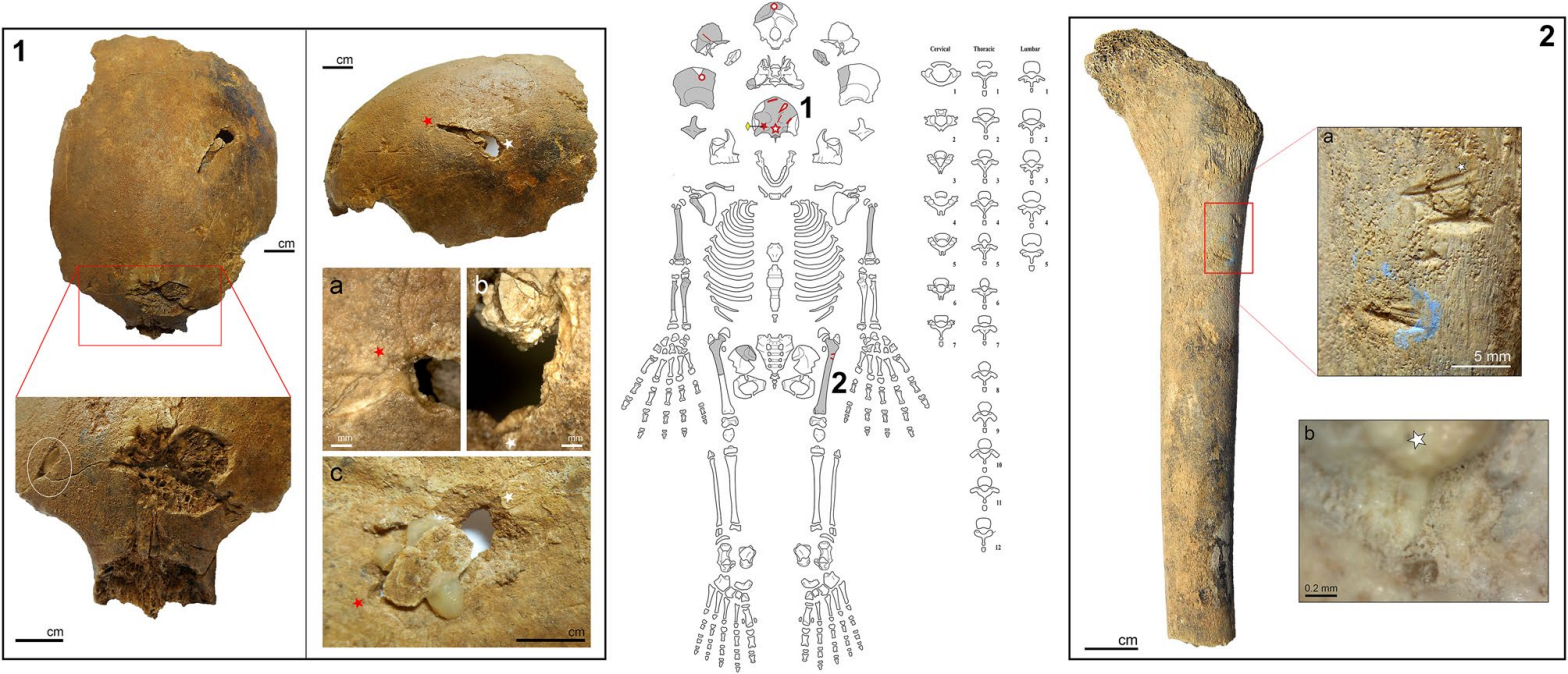
Location and images of the observed osseous lesions on JS 14. Center: schematic scheme of JS 14′s skeletal preservation. Grey parts represent preserved bones, star = blunt force trauma, full star = unhealed puncture, open circle = perforations, yellow diamond = embedded artefact in a puncture, dash = drags traces of projectile impacts, line = cutmark. Box n°1: lesions of the frontal bone on JS 14. Left: superior view of the frontal bone with, below, the magnification in frontal view of the red box showing the blunt force trauma and the embedded lithic (white oval) with hinge fractures. Right: left lateral view of the frontal bone displaying the projectile perforation. Red and white stars are reference points for the magnified area; a = hinge fractures at the level of the entrance of the projectile; b = crushing fractures on the border of the perforation; c = endocranial view of the internal beveling. Note the miss-glued piece of bone associated to the perforation, part of the original conservation works. Box n°2: Projectile impact marks on the left femur of JS 14. Left: anterior view of the preserved part of the left femur. a = close up on the two set of drag marks located on the antero-lateral side of the shaft. White star put as reference point for the magnified area. b = detailed view of the superior drag revealing the wide flat bottom of the groove and the parallel microstriations (magnification ×245).

The first case concerns the double burial of two children JS 13 and JS 14, who are close to 5 and 4 years of age, respectively, based on dental development and bone growth. Five lithic artefacts were found in association with the two individuals (^23^, p. 963). Although no osseous lesion was visible on JS 13, both the cranium and infra-cranium of JS 14 have unhealed trauma caused by projectile impacts (Fig. 2). The majority of the lesions are located on the calvaria and none had previously been documented. The frontal bone exhibits a blunt force trauma at the level of the glabella. Several drag marks and an oblong perforation are also present on the left side of the frontal squama, as well as scraping drag marks close to bregma. Both a puncture site with faulting and part of an embedded artifact are visible approximately one centimeter above the left orbit (Fig. 2-1). A perforation is also present on the right parietal and on the occipital. The frontal and occipital perforation exhibit internal bevelling consistent with projectile impacts^34^. A further set of marks is visible on the left femur, including two groups of drags on the antero-lateral border of the proximal part of the diaphysis (Fig. 2-2). The first group has two subparallel incisions with wide flat floors marked with parallel microstriations. Bone flaking is also present at the end of the trajectory. The second drag is located about one centimeter below the proximal one, and oriented slightly more anteriorly, with a bisecting pattern at the end of the marks. Based on these cutmark characteristics, the projectile most probably arrived from the medial side of the femoral diaphysis, in a downwards motion and towards the lateral side.

**Figure 3:**
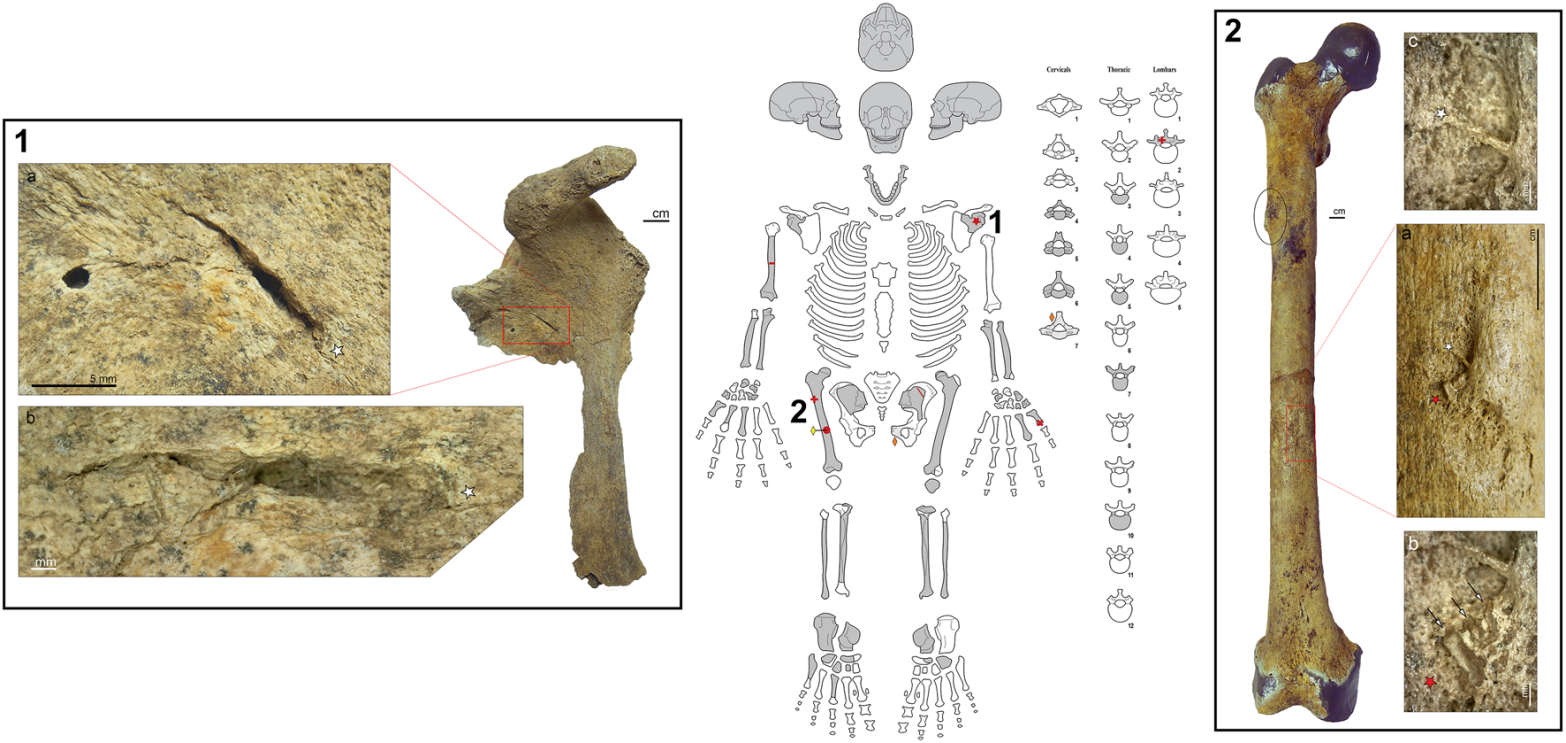
Location and images of the observed osseous lesions on JS 31. Center: schematic scheme of JS 31′s skeletal preservation. Grey parts represent preserved bones, striped areas are missing bone or areas, full star = unhealed puncture, dash = drags traces of projectile impacts, line = cutmark, plus sign = healed lesions, time sign = healed fracture, full circle = healed puncture, yellow diamond = embedded artefact in a puncture, orange diamond = embedded artefact in lost bone. Box n°1: Projectile impact puncture on the left scapula of JS 31. a = red rectangle close up on the subscapular fossa showing the puncture associated with flaking and faulting. b = composite microscopic image of the puncture displaying the crushing of the bone in the lower border of the puncture (magnification ×40). Box n°2: Healed lesions on the right femur of JS 31. Red rectangle = healed projectile lesion, black ellipse = bone callus. a = red rectangle close up of the healed projectile injury with red and white stars as reference points for the magnified area b and c. b = microscopic view of the three embedded lithic chips marked by arrows. c = microscopic view of a bony bridge separating two geometric marks indicating the presence of two lost lithic chips (magnification ×50).

The second case, JS 31, focuses on the remains of a probable male over 30 years old based on his heavy dental wear and bone remodeling. Seventeen lithic artefacts found in situ were in direct association with his skeletal remains, with two embedded in the bone and fifteen within the physical space of the body (^23^, p. 973–974). The embedded chips were originally found in the seventh cervical vertebra and in the left pubis (^19,23^), with the bone around both lithics showing severe reactive changes (cf. p. 989 in^23^, and p. 1027 in^19^). Unfortunately, these bones are not part of the collection donated to the British Museum. The lesions observed on JS 31 are located on the infra-cranial skeleton. Our reassessment revealed previously unidentified healed and unhealed projectile impact marks, as well as healed lesions that are most probably the result of earlier interpersonal injuries. The new unhealed PIMs identified include a puncture with crushing, faulting and flaking of the bone surface on the anterior part of the left scapula (Fig. 3-1) and a deep V-shaped drag (2 cm long) on the posterior-medial side of the humerus. JS 31 also has a healed fracture of the distal extremity of the right first metacarpal. Finally, the right femur offers further evidence of healed lesions, with the presence of a bone callus on the lateral side of the proximal part of the shaft and of a healed projectile wound on the anterior side at midshaft. Three previously unidentified embedded lithic chips were found trapped in the healing bulge of the latter (Fig. 3-2).

**Figure 4:**
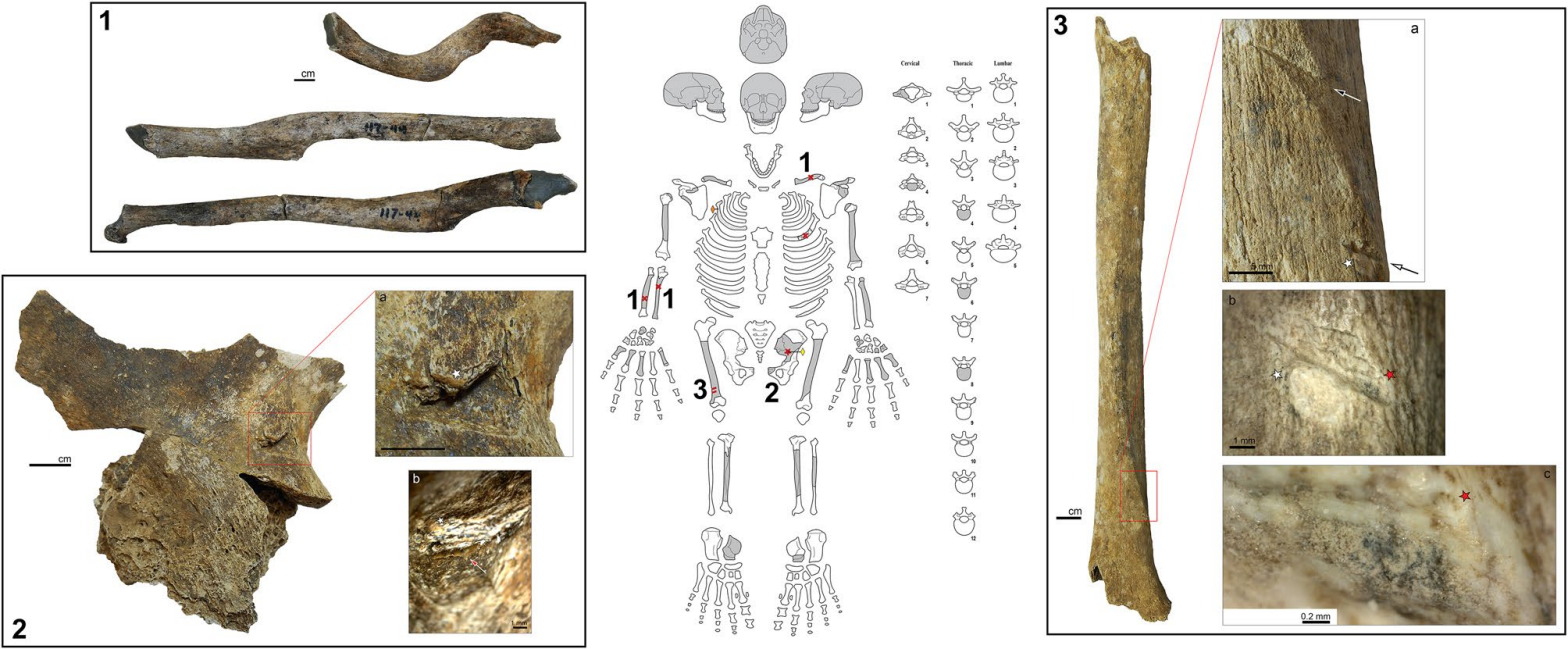
Location and images of the observed osseous lesions on JS 44. Center: schematic scheme of JS 44′s skeletal preservation. Grey parts represent preserved bones, striped areas are missing bone, crisscross areas are bone whose exact anatomical position is unknown, full star = unhealed puncture, dash = drags traces of projectile impacts, time sign = healed fracture, yellow diamond = embedded artefact in a puncture, orange diamond = embedded artefact in lost bone. Box n°1: Healed fractures on JS 44. From top to bottom, left clavicle superior view, right radius anterior view and right ulna anterior view. Box n°2: Lateral view of the left pelvis of JS 44 with a projectile impact puncture with an embedded lithic flake. a = red rectangle close up of the PMI with white star as reference point for the magnified area b. b = microscopic view of the puncture showing the laminated aspect of the superior border and the lithic artefact inside the puncture indicated by the red arrow (magnification 30x). Box n°3: Double parallel drags on JS 44 located on the posterior surface of the right femur diaphysis, at the level where the lateral supracondylar line, which delimitates the lateral part of the popliteal plane, meets to lateral side of the femoral diaphysis. a = red rectangle close up showing the two parallel drags and the direction of the projectile with the arrows. White star as reference point for the magnified area b. b = microscopic close up on the distal drag showing the flaking of the superior border at the origin of the drag. Red star as reference point for the magnified area c (magnification ×45). c = composite microscopic view of the proximal part of the distal drag displaying the wide flat bottom of the groove and the parallel microstriations (magnification ×235).

The third case, JS 44, are the remains of a possible female that appears to have been older than 30 years. Twenty-one lithic artefacts were found in close association with the skeleton, one of which was embedded in the fourth rib (^23^, p. 978). Wendorf also noted two examples of chip and/or flake alignments during the excavation which he interpreted as evidence of composite projectile use^23^. The fourth rib with embedded “backed flake” is, unfortunately, also not present in the British Museum Wendorf collection. As with JS 31, all the lesions observed on JS 44 are located in the infra-cranial skeleton (Fig. 4), with healed fractures present on the left clavicle, right ulna and radius, and one left rib. The fracture of the left clavicle shaft, located on the acromial end of the diaphysis, reveals a slight torsion and a displacement of the bone fragments. The right forearm healed fracture is oblique, with a displacement (translation and rotation) of the two broken pieces (Fig. 4-1). The clavicle and forearm fractures most probably occurred during the same event. Given the oblique nature in the forearm and acromial involvement in the clavicle, they may have been caused by an indirect trauma, such as a bad fall, rather than a defensive parry fracture (see^33^). The other lesions, however, are clearly the result of projectile impacts. A triangular notch on the lateral face of the ilium, about 1 cm from the greater sciatic notch, has a lithic fragment embedded in the incision. The laminated aspect of the bone overlying the flake suggests there was an attempt to extract the projectile (Fig. 4-2). The morphology of the PIM also indicates the projectile travelled from the postero-medial to the antero-lateral side of the left pelvic bone, which implies the projectile was travelling back to front. PIMs were also observed on the right femur. Two parallel drags less than 1 cm long and approximately 2 cm from each other are visible on the posterior side of the diaphysis. These drags exhibit a flat bottom with parallel microstriations. The most distal one shows flaking marks on the proximal border (Fig. 4-3). Significantly, the angle of penetration into the bone differs for both drags, with the most proximal one being more tangential. These drag marks reflect a projectile trajectory that came from the disto-lateral to the proximo-medial part of the bone. This upward direction suggests the individual was hit while running or that the projectile was drawn from a lower position. Finally, the spacing between these two drags and their morphology are consistent with the penetration from a single composite projectile. This hypothesis is strengthened by Wendorf’s field observation of in situ lithic alignments associated with JS 44.

### Burial selection and mortality profile

In view of the high number of individual with evidence of interpersonal violence, the frequency of projectile impact marks, and the presence of several double or multiple burials, the site’s mortality profile was analyzed to investigate possible patterns in burial selection (see^35,36^). Should the cemetery reflect a single warfare event, an unbalanced demographic profile (e.g. the overrepresentation of a certain sex or age group less likely to die otherwise) is probable^37^. At Jebel Sahaba, the individuals whose sex could be assigned (n = 39) revealed no bias, with 48.7% females and 51.3% males. The age distribution shows a underrepresentation of non-adults ([< 20] = 29.5%) compared to the theoretical percentage ([< 20] = 54.5% ± 9.5%) for a pre-jennerian population with a life expectancy at birth of between 25 and 35 years^38^. However, this imbalance is mostly due to the lack of perinates, neonates and young children (age cohorts [0–1] and [1–4]) whose mortality quotient stands outside the lower limits of the theoretical values (cf. Supplementary Fig. S4). The small proportion of very young children is not unusual in pre-Neolithic funerary assemblages and may relate to demographic factors, cultural behaviors such as the separate burial of young infants, or poor preservation, although the latter is unlikely at Jebel Sahaba^39–42^. To account for these possibilities, we focused on the age cohorts over than five years old to assess any biases in age-at-death representations^40,43^. Both the J:A ratio (JS = 0.200) and the mean childhood mortality value (JS = 0.073) are below the threshold of biased cemetery populations (respectively J:A < 0.380 and MCM < 0.135;^43^). In the event of a mortality crisis linked to a single event, demographic anomalies are usually found in age cohorts less likely to die otherwise (namely the [10–14] and [15–19] cohorts) by way of an overrepresentation in their mortality quotient^35,36,44^. The Jebel Sahaba cemetery mortality curve does not include such an anomaly.

### Reassessment of the lithic assemblage

We identified 13 new pieces embedded in projectile impact marks on the human remains and counted the multiple fragments found in one PIM as one artefact. Based on these findings, a new total of 132 artefacts were found in direct association with 28 individuals (cf. Supplementary Table S3). With the exception of a few flakes and points, the artefacts collected at the surface of the site (n = 72) differ in terms of their typology and the raw material used when compared to the ones found inside the burials and within the physical space of the skeletons (n = 115; Supplementary Text S3 and Fig. S5).

Our reexamination confirms the diversity of artefact shapes with a tendency toward small size pieces. Despite a strong typological variability, most lithic artifacts found inside the burials can be identified as projectiles or armature elements, including the unretouched parts. Significantly, preliminary functional analysis shows that some artefacts have impact fractures. Technological and typological elements fit well with the definition of the Qadan industry^23,45,46^. The current reassessment also revealed strong similarities to the Tushka 8905-B industry unambiguously attributed to the Qadan (^45–47^; Supplementary Text S3).

Based on this reanalysis, almost half of the elements used as weapons are unretouched flakes and micro-flakes that, as noticed by Wendorf^23^, would have been missed in any other context (cf. Supplementary Fig. S5). In the case of Jebel Sahaba, their association to weaponry is indisputable. Most appear to be laterally shafted composite elements used as part of projectiles. The points would have been mounted at the end of shafts, with crescents laterally shafted. Their diversity in both size and shape suggests the use of several types of weapons, particularly light arrows but also much heavier arrows or spears. Finally, the use of points with oblique or transverse distal cutting edges appears to indicate that one of the main lethal properties sought was to slash and cause blood loss. The fact that many were found inside the volume of the skeleton also indicates their efficiency at penetrating the body. Those found at the site are likely to be the ones that had detached themselves from their shaft and not successfully removed prior to burial.

## Discussion

Since its discovery in the 1960′s, the Jebel Sahaba cemetery has been regarded as the oldest evidence of organized warfare caused by environmental constrains e.g.^24–28^. However, the lesions observed on the Jebel Sahaba skeletons and the nature of the funerary complex had not been reassessed and it remained unclear whether the site was the result of a single conflict, a specific burial place or the evidence of sustained inter-personal violence in Late Pleistocene hunter-gatherer groups^18^.

Wendorf^23^ and Anderson^19^ had highlighted the projectile nature of several lesions, particularly those with embedded lithic artefacts. Here, macroscopic and microscopic methods were used to distinguish projectile injuries from slicing cutmarks and taphonomical modifications (see^34,48–52^). More than half of the individuals with lesions buried at Jebel Sahaba (n = 41) exhibit clear projectile impact marks (61.0%, n = 25), and most show signs of trauma (92.7%, n = 38). Irrespective of age and sex, the majority of those buried at the site exhibit signs of interpersonal violence that involve projectile weapons. The number of individuals with both healed and unhealed traumas also increases with age from adolescence (n = 1), to young adults (n = 2) and adults (n = 13). Importantly, the co-occurrence of ante-mortem and peri-mortem lesions on several Jebel Sahaba individuals had not previously been noted and indicates that some had experienced multiple episodes of interpersonal violence during their life.

As with experimental studies on ungulates^53,54^, drag marks are the most frequent PIMs observed at Jebel Sahaba followed by punctures, particularly on the appendicular skeleton. Experimental work also reveals that 45.0% of ungulates PIMs include microscopic fragments of the actual weapons that end up embedded in the bones, either at impact or while attempting to remove the weapon (^34,53^). At Jebel Sahaba, artefacts were found in one third of the drag and puncture impact marks (31.3%, n = 20). Of these, the great majority were in puncture marks (70.8%, n = 17). The PIMs patterns supports the use of composite weapons made of shafted retouched and unretouched flakes, including light and heavy projectiles. This is corroborated by the alignment of flakes and chips within the physical space of the skeletons, the reassessment of the lithic assemblage and cases of parallel drags less than 2 cm apart consistent with ethnographical and experimental spear and arrow shaft diameters^54–56^.

Identifying interpersonal violence on skeletal remains is not always straightforward and often relies on the type of trauma and the archaeological context^57,58^. Clear examples of fatal interpersonal blunt and sharp force trauma go as far back as the Middle Paleolithic^59,60^, while the oldest Palaeolithic projectile trauma with an embedded point date to the Epigravettian period^61^. Based on the available evidence, the number of projectile injuries appears to increase over time and cases of fatal trauma in Europe become more frequent during the Mesolithic^62^. In Africa, the site of Nataruk provides the closest parallel of inter-personal violence to Jebel Sahaba^63^. Situated west of Lake Turkana and dating to around 10.5–9.5 ka, the individuals found in Nataruk appear to exhibits signs of violent death through projectile impact marks (punctures and perforation), sharp and blunt force trauma and fractures. Although this evidence has been debated^64^, the Nataruk example also differs from Jebel Sahaba in that there is no clear pattern of deliberate burial, no signs of trauma on children and a lack of healed trauma in the adults.

Violent behavior in past and present hunter-gatherer societies appears to vary, in part reflecting the period, culture and the level of organization of mobile and semi-sedentary societies e.g.^24,25,65^. Several ethno-archaeological examples suggest that the concept of warfare can encompass all form of antagonistic relationships from feuds, individual murders, ambush attacks, raids and trophy taking to bloody clashes and larger armed conflicts^25,27,65^. The level of warfare can vary, with some conflicts being all-encompassing, constant and deadly, while others are episodic events of various intensity that occur sporadically^65^. At Jebel Sahaba, the co-occurrence of healed and unhealed lesions strongly supports sporadic and recurrent episodes of interpersonal violence between Nile valley groups at the end of the Late Pleistocene. The projectile nature of at least half of the lesions suggests inter-group attacks, rather than intra-group or domestic conflicts^65–69^, and the frequency of healed wound confirm that these events were not always lethal and could occur several time during the life of an individual. While the number of parry fractures is higher among female individuals, and the blunt force trauma mostly present on immature individuals, the remaining pattern of lesions on female and immature individuals at Jebel Sahaba is inconsistent with domestic violence^70,71^.

A catastrophic single mass burial is highly unlikely and not supported by the archaeological evidence and the demographic analysis. With the exception of a higher percentage of parry fractures in females, there appears to be no patterning in the distribution of trauma or PIMs by rather age or sex. Based on the lesions, the projectile direction also reveals an equal number of posterior and anterior strikes that do not support face-to-face battles. Rather, the involvement of a range of ages and both sexes, with primary (n = 26), double (n = 4) and multiple (n = 4) burials, including some with evidence of disturbance due to the addition of later individuals^23^, indicate small episodes of recurring violent events such as raids or ambushes against this community. This appears to have taken place on a short timescale given the homogeneity of the burial place and practices.

Special burial places for the victims of violence are documented in ethnological and historical records^72^. At Jebel Sahaba, the percentage of individuals with traces of peri-mortem traumas and/or lithic artefacts found within the physical space of the skeleton is 54%. If multiple burials are treated as simultaneous deaths and individuals without detectable signs of a violent death but buried in direct association with others that have are included, the percentage is closer to 64%. The nearby site of Wadi Halfa (6-B-36) does not seem to document comparable levels of violence as the percentage of individuals with traumas (22.2%, n = 8) is lower than at Jebel Sahaba (62.3%, n = 38). However, an unhealed projectile trauma with an embedded stone point in a cervical vertebra is documented^21^, and the frequency of the most identifiable lesion in Wadi Halfa, healed parry fractures, is similar to Jebel Sahaba (respectively 8.3%, n = 3, and 9%, n = 6). Therefore, we consider more likely that the level of interpersonal violence observed in Jebel Sahaba reflects broader inter-group behavioral relationships in the Nile valley at the end of the Late Pleistocene rather than specific funerary practices.

The high level of interpersonal violence observed at the site may, in part, have been driven by the climatic variability. During the Late Pleistocene, few human remains are recorded in the Nile valley. This is mirrored by a drastic reduction in the archaeological record with little evidence for the presence of humans along the lower Nile from Marine Isotopic Stage 4 (~ 71 ka) to the Last Glacial Maximum^9^. During this time period, the survival of small groups in the fewer sustainable areas in Upper Egypt and Lower Nubia is supported by the unusual phenotypic diversity, probably related to population fragmentation and isolation, found in the Late Pleistocene fossils of this region^19,21,73–75^. With variation of lithic industries indicating different cultural traditions and the co-occurrence of large cemetery spaces suggesting some level of sedentism^15^, severe territorial competition between the region’s hunter-fisher-gatherer groups is likely to have occurred when forced to adapt to the drastic environmental changes recorded at the end of the Last Glacial Maximum and the beginning of the African Humid Period (cf. Supplementary Text S1). Climate change is most likely to have been a driver towards a violent competition for resources over time as documented in the ethno-archaeological record e.g.^25,65,68^.

## Conclusions

For the first time since Wendorf’s original publication^11^, a complete reassessment of the Jebel Sahaba cemetery was used to clarify the nature, extent and dating of the violence experience by the individuals buried at the site. First, direct radiocarbon dates, between 13.4 and 18.2 ka, confirm the antiquity of the site, making Jebel Sahaba the oldest cemetery in the Nile valley. Second, using modern approaches and methods, our reappraisal undeniably supports the interpersonal nature of the lesions and confirms the projectile origin of most of the trauma. Our analyses also show that out of sixty-one individual, 26.2% of had signs of perimortem traumas and 62.3% displayed healed and/or unhealed traumas (excluding undiagnosed lesions) regardless of the age-at-death or sex, including children as young as 4 years old. Third, the reassessment of the lithic artefacts associated to each burial reveals that most were elements of composite projectile weapons. Fourth, although double and multiple burials are present, most probably indicating simultaneous deaths, demographic data and burial disturbance caused by subsequent interments does not support a single catastrophic event. While acknowledging the possibility that the Jebel Sahaba cemetery may have been a specific place of burial for victims of violence, the presence of numerous healed traumas and the reuse of the funerary space both support the occurrence of recurrent episodes of small scale sporadic interpersonal violence at the end of the Pleistocene. Most are likely to have been the result of skirmishes, raids or ambushes. Territorial and environmental pressures triggered by climate changes are most probably responsible for these frequent conflicts between what appears to be culturally distinct Nile Valley semi-sedentary hunter-fisher-gatherers groups.

## Materials and methods

### The British Museum Jebel Sahaba collection

In 2001, Wendorf donated all the archives, artefacts and skeletal remains from his 1965–1966 Nile Valley excavations to the British Museum^76,77^. Judd’s preliminary osteological analysis noted discrepancies between field notes, photographs and associated skeletal remains, including the absence of three individuals, JS 1, JS 3 and JS 30, as well as some of the bones with embedded lithic artefacts described by Anderson^19,76^. Not part of the British Museum donation, their whereabouts remains uncertain. The three missing individuals could not be included in this reanalysis. Regarding the few missing bones of reassessed individuals, we relied on Anderson’s description of the trauma as they could not be examined microscopically. Judd’s survey of the skeletal remains also noticed the presence of extra bones or teeth from additional individuals. Excluding the remains of the three missing individuals, our reanalysis also found supernumerary bones and teeth and, with the British Museum collection, the site can now be regarded as including the remains of at least 64 individuals, three of whom are now missing.

In addition to the lithic assemblage from the fill around the skeleton that we attributed to the surface find assemblage (n = 72), our reassessment included 115 pieces from the original collection described as directly associated to the skeletons. Three pieces from burials JS 25, JS 45 and JS 47 are not in the British Museum collection. A supplementary piece was, however, found associated to burial JS 26. This piece was probably mixed with the surface material early on, which could explain its absence in Wendorf’s inventory (although the piece was drawn in p. 987 in^23^). We also included five pieces found near burials JS 101 to JS 107. Although not directly in contact with the skeletons, their association to the individuals of this multiple burial is suggested by Wendorf (^23^, p. 988). These artefacts were part of our reassessment but we remained cautious as to their association with the burials.

### Biological identification

The analysis involved a full reevaluation of the age and sex using the latest anthropological methods. In some individuals, assessments were limited by the state of preservation and completeness of the skeletal remains. Biological sex was based on the morphology and dimensions of the pelvis^78–80^. When the pelvis was not sufficiently complete, the cranium and mandible were also used^81^ to assign sex preceded by the letter “p” for “probable” (i.e. pM = probable Male). When cranial morphology was the only method available, a question mark was added to denote the limitation of the approach (i.e. pM? = possible Male). Finally, when the cranium and the pelvis were absent, individual are classified as undetermined (UND). The age-at-death of the immature individuals is predominantly based on the stage of dental development following Moorrees et al*.*^82,83^. In the rare occasions where teeth were not present or preserved, the state of skeletal growth and development were used^84–86^. In adults, Schmitt^87^ was employed to score the remodeling of the iliac sacro-pelvic surface (ISPS). Given the strong dependence of the senescence processes on population, environmental and behavioral factors^88^, when the ISPS was not preserved, we chose to cautiously assign the mature individuals into the following broad age groups based on the level of dental wear ([> 20 years] = individual with dental wear below category 4; Molnar^89^; [> 30 years] = individual with dental wear above Molnar’s category 3). In the rare instances where dental remains were absent, mature individual were designated as adults [> 20 years] if no sign of join remodeling or entheseal changes where observable. In all the other cases, the individual was assigned to the age group [> 30]. We used the J:A ratio between immature individuals aged [5–14] and adults individual over 20 years [> 20] and the mean childhood mortality value (MCM) to address potential bias in the Jebel Sahaba cemetery population^40,43^. In order to discuss potential demographic anomalies in the Jebel Sahaba cemetery, we grouped the individuals in six conventional age cohorts ([0– < 1], [1–4], [5–9], [10–14], [15–19] and [20–29 years]) that allow for comparisons with theoretical mortality values of a population with a life expectancy at birth of between 25 and 35 years^38^. Immature individuals falling into two cohorts based on age-at-death estimate standard deviations were assigned to the most probable one according to Sellier^35^.

### Lesions and Projectile Impact Marks (PIMs) characterizations

Extensive and detailed microscopic analyses of the all areas exhibiting taphonomic and/or anthropogenic traces were conducted using a digital microscope (Dino-Lite Premier) with a 5 Megapixels resolution, a polarizer and a 30×–250× magnification range. Following the recommendations of Smith et al*.*^34^, each potential lesion was checked for embedded lithic fragments and characterized. Non-anthropogenic traces, mainly due to gnawing and termite activity, were differentiated using macroscopic and microscopic criteria^48,49,52^. The Jebel Sahaba individuals were buried in pits, filled by sediment and covered by sandstone slabs (see Supplementary Text S2), and although trampling marks were unlikely, the diagnostic criteria from Domínguez-Rodrigo et al*.*^50^ were used to exclude such taphonomic changes.

Projectile Impacts Marks (PIMs) were characterized using projectile bone damage identification criteria derived from experimental archaeological research^34,51,53–55,90^. Although based on the hunting of small and large ungulates, these experimental studies provide a clear system of projectile trauma classification that is often lacking in analyses of interpersonal violence^34^. The terminology and classification used in this study are characterized by the level of hard tissue projectile penetration defined by O’Driscoll & Thompson (^51^, see Supplementary Text S4 and Fig. S6). In a number of cases, the projectile origin of a lesion could not be identified, sometimes due to poor preservation and uncharacteristic changes, and the term trauma is used. It also covers all the healed or unhealed bone fractures, blunt force trauma and perforations with no PIM signs. The term fracture is defined as a partial or complete break in the continuity of a bone^33^. Finally, the term lesion refers to an injury whose nature or anthropogenic origin could not be determined. The presences of bone callus or abscesses were also recorded. Signs of new bone formation or remodeling linked to healing processes were carefully noted and classified as healed, implying a delay of at least three weeks between the injury and death (^33^; Supplementary Fig. S2).

## Supplementary Information

Supplementary Information.
