# Supplementary material for: New insights on interpersonal violence in the Late Pleistocene based on the Nile valley cemetery of Jebel Sahaba
Source: Sci Rep. 2021 May 27;11:9991. doi: 10.1038/s41598-021-89386-y (PMC8159958; doi:10.1038/s41598-021-89386-y)
Supplement: Supplementary file 1 — Supplementary Information. [file 41598_2021_89386_MOESM1_ESM.pdf]

Supplementary Information for

New insights on interpersonal violence in the Late Pleistocene based on the Nile valley cemetery of Jebel Sahaba

Isabelle Crevecoeur[1*], Marie-Hélène Dias-Meirinho[2], Antoine Zazzo[3], Daniel Antoine[4], François Bon[2].

[1] UMR 5199-PACEA, CNRS, Université de Bordeaux, Allée Geoffroy Saint-Hilaire, CS 50023, 33615 Pessac Cedex, France.
[2] UMR 5608-TRACES, Université de Toulouse Jean Jaurès, Maison de la Recherche, 5 Allées Antonio Machado, 31058 Toulouse Cedex 9, France.
[3] UMR 7209-AASPE, CNRS, MNHN, CP 56 – 43 rue Buffon, 75005 Paris, France.
[4] Department of Egypt and Sudan, The British Museum, Great Russell Street, London WC1B 3DG, UK.

***Corresponding author :**
Isabelle Crevecoeur,
UMR 5199 PACEA, Université de Bordeaux,
B8, Allée Geoffroy Saint-Hilaire, CS 50023,
33615 Pessac Cedex, France.
isabelle.crevecoeur@u-bordeaux.fr

This file includes:

Supplementary Text p. 2-9
- Text S1: Environmental and archaeological contexts p. 2-4
- Text S2: The Jebel Sahaba cemetery: context and dating p. 5-6
- Text S3: Description of the lithic assemblage p. 7-8
- Text S4: Projectile Impact Marks (PIMs) terminology p. 9

Supplementary Figures S1 to S6 p. 10-15
Supplementary Tables S1 to S3 p. 16-20
Supplementary references (1-83) p. 21-26

SI Text S1: Environmental and archaeological contexts

In Africa, geological evidence points to generally dry conditions during the Last Glacial Maximum (LGM, ~23-18 ka; 1). The LGM was then followed by the African Humid Period (~15-5.5 ka) which ended abruptly with the second half of the Holocene and the onset of more arid conditions (2). However, these climatic variations were anything but gradual and stable. At a large scale, the African Humid Period was punctuated by short arid episodes reflecting the Holocene's high climatic variability (3). Integrated analyses of well-dated archaeological settlements and fluvial units reveal hydrological fluctuations and monsoonal changes, engendering climatic and environmental constraints that appear to have impacted human settlements along the Nile valley during the last 25 ka (4-12).

During the Marine Isotopic Stage 2 (MIS 2; 27.8 - 14.7 ka; 13), the Nile Valley probably functioned as a refuge for human populations. This is attested by the high density of archaeological contexts in Upper Egypt and Lower Nubia dated to the aridity peaks of the LGM and the second part of the Heinrich Stadial 1 (HS1b; 16-14.5 ka; 10, 12, 14-16). The Blue Nile and the White Nile were then highly seasonal rivers and the Sudd swamps had yet to develop (14, 17). The abrupt changes in the Nile flow regime that occurred around 15-14 ka, with the return of the summer monsoon in intertropical Africa, produced an overflow of the Lake Victoria in the Ugandan headwaters of the White Nile (6-7, 12). This period of hydrological instability lead to the (re)establishment of the present Nile-flow regime and caused severe flooding as far as the Egyptian part of the Nile valley (6, 18). This Wild Nile episode was interrupted by a short-term return of drier and colder climatic condition that coincides with the Younger Dryas interval (YD; ~12.9-11.7 ka; 19). The incision of the Nile during the YD probably led to equivalent challenges in human adaptation as the preceding high floods (20). It is only after the YD that the monsoon conditions of the African Humid Period become more stable.

If traces of human occupations are extremely sparse during the Late Pleistocene in the Nile Valley, an increase in population density documented in Upper Egypt and Lower Nubia during the MIS 2 may have benefited from the creation of lakes by eolian dunes acting as dams during the reduce activity of the Blue Nile and White Nile (6, 10, 20-21). The archaeological sites from this period are restricted to the floodplain in both Upper Egypt and Lower Nubia (4-5, 10, 15-16, 22) (**Fig. S1**). Historical factors may also have led to some geographical bias and influenced the density of recorded sites. In Lower Nubia, this appears to have been in part a consequence of the extensive prehistoric survey prior to the construction of the Aswan Dam, as well as other geological factors affecting site preservation. However, the quasi absence of archaeological sites in Lower Egypt and Upper Nubia during this period is striking (10, 16, 22-23). More than around a hundred of archaeological sites have been documented so far in the Upper Egypt and Lower Nubia region for the final part of the Late Pleistocene period (~25-11.7 ka; 16; **Fig. S1**). They are associated with a variety of lithic assemblages that linked to Late Paleolithic, or Late Stone Age, industries based on the production of flakes and elongated products of small dimensions, including high proportions of backed tools (16, 22). The great variability of typological characteristics of these lithic assemblages has led to the recognition of different industries that seem to be regionally and

chronologically constrained, with some industries occurring only in Lower Nubia or in Upper Egypt (*cf.* **Fig. S1**). For the period between 20-11 ka that encompasses the chronological range of the Jebel Sahaba cemetery (*cf. infra*, Supplementary **Text S2** and Supplementary **Table S1**), the most represented and well-dated complexes are the Kubbaniyan, the Ballanan-Silsilian, the Isnan, the Halfan and the Qadan (22, 24-27; *cf.* **Fig. S1**). Archaeozoological analysis of these Late Pleistocene settlements indicate similar human subsistence strategies based on the exploitation of the Nilotic environment (22). Seasonally adapted fishing activities, shellfish consumption, medium and large ungulate hunting and plant tuber processing are documented at various locations (22, 28-30). Finally, the existence of sophisticated figurative rock art, although not correlated to any lithic industry so far, is also attested at Qurta around 15 ka (31). In this framework of limited subsistence strategy options and constrained geographical habitable environment, the development of a wide range of lithic cultural traditions is though-provoking. If differences in raw material availability on either side of the first Cataract could be accounted for, or the pertinence of some of the criteria used to defined these lithic industries (as well as the integrity of some assemblages) questioned, the relevance of these chrono-cultural units has rarely been challenged at a regional scale (16, 18, 22, 32-35). It has therefore been suggested that each of these lithic entities could represent a cultural tradition reflecting the structuration of human group identity in this restrained habitable area (22). The concomitant occurrence of large graveyards, among the oldest for the Late Pleistocene in Africa, in such a confined area reinforces the hypothesis that these represent strong social units of residential groups (36). After the last aridity peak of the HS 1b (~ <14.5 ka) and before the stabilization of more humid conditions (~ >11 ka), the number of dated archaeological sites decreases in Lower Nubia and, in Upper Egypt, such sites seem to disappear completely (15-16). This period witnessed catastrophic environmental changes owing to Nile flow regime changes that may have impacted the survival of humans in the Nile Valley (5, 10, 16, 20).

With the stabilization of more humid conditions during the beginning of the Holocene (~10.5-7.3 ky), prehistoric settlements moved to the Eastern Sahara as it turned into a savannah-like environment and probably became more hospitable than the Nile valley (4-5, 37-39). No precisely dated archaeological sites are documented in the Egyptian Nile Valley during the Early Holocene but periodic traces of human presence, probably linked to seasonal activities, are suspected (5, 40-41). The only two regions that have yielded archaeological evidence in the Nile Valley during the Saharan exodus have been associated to Mesolithic human occupations in Central Sudan and Upper Nubia (*e.g.* 8, 42-44). The subsequent decline in human occupation in the Sahara from 7.3 ky to 5.5 ka relates to the cessation of regular monsoon rains that forced human populations to relocate to new ecological niches and/or to begin the migration to the Sudanese plains and the Egyptian Nile valley (4-5, 36, 39-40).

In parallel with these climatic fluctuations, the beginning of the Holocene witnessed the appearance of new subsistence strategies with the apparition of the oldest-known form of pottery in the Sahara and the Nile Valley (~ 10.5 ka; 45-46), as well as the emergence of herding activities (47-50). The origin and spread of pastoralism in North Africa is debated with circumstantial evidence of early cattle domestication reported at Nabta Playa and Bir Kiseiba about ~10-8 ka (51-53) and the oldest occurrence of bones from domestic ovicrapines documented in the Red Sea Mountains and in the western desert from ~8.2 ka onwards (54-56). While changes in subsistence strategies emerged in

the western desert and the Red Sea Mountains at the beginning of the Holocene, they are not related to a fundamental break in the hunter-fisher-gatherer traditions since, in many of these contexts, hunting of wild mammals and gathering of wild seeds were still dominant food procurement activities (48, 56). Only in the second half of the Holocene (after ~7.0 ka), when monsoonal rains decrease and populations resettle in the Nile valley, do we witness Northeast African populations taking up truly pastoral ways of life (5, 47, 57-59).

SI Text S2: The Jebel Sahaba cemetery: context and dating

The site of Jebel Sahaba (site 117), now submerged underneath the lake created by the Aswan High Dam, is located about 3 km north of the modern town of Wadi Halfa. While in use, the cemetery was located one kilometer east of the ancient shore of the Nile (60). The site was discovered as part of the UNESCO-funded salvage campaigns of the sites that were to be flooded by the construction of the Aswan High Dam (60). Paepe and Perkins, part of the Columbia University Nubian Expedition initially documented the site in 1962 (61). The individuals associated with this first excavation are referred as JS C-1, JS C-2 and JS C-3 in the Jebel Sahaba collection (62). In 1965, within the framework of the Southern Methodist University field season, Wendorf visited the site and further tested the areas immediately adjacent to the first excavation (63). The successful recovery of additional human remains led to a full-scale excavation and forty-nine skeletons (JS 1 to JS 49) were uncovered in 1965, with an additional six excavated in 1966 (JS 100 to JS 107) under the supervision of Dr. Marks (60).

The northern part of the cemetery was stripped by erosion, revealing disturbed and heavily cemented human remains. The rest of the cemetery consisted of well-preserved skeletons buried in oval pits that were cut into a weakly cemented sediment and covered by thin sandstone slabs (60). Most were primary individual burials, with some double and multiple interments, as well as secondary deposits caused by later burials (36). In total, sixty-one skeletons were recovered, with most individuals carefully buried in contracted position on their left side, with the head toward the east, facing south. In most cases, the hands were positioned close to the face and the lower limb was flexed with the feet close to the pelvis (60).

Although no occupation deposits were found in the vicinity of the cemetery, more than a hundred lithic artefacts were found inside or around the burials. The artefacts directly associated to the burials show strong resemblances with the Qadan lithic industry, particularly specific tool types such as crescent-like backed pieces (60; Supplementary Text S3). Most pieces are unretouched flakes and chips that would, in a different context, be identified as debitage artefacts rather than tools. In the case of Jebel Sahaba, their association to weaponry is indisputable (supplementary Text S3). It may stem from an opportunistic or planned use of the cutting edge, suggesting highly flexible cultural behaviors according to Wendorf (60, 64).

The Qadan lithic industry is documented in Lower Nubia from the end of the Late Pleistocene until the Holocene with radiocarbon dates ranging from ~12.0-20.2 ka (16, 22, 34; *cf.* **Fig. S1**). The antiquity of Jebel Sahaba and its pene-contemporaneity with the Qadan industry was confirmed using ten direct radiocarbon dates carried out on five individuals from the cemetery (36, 65; **Table S1**). The oldest date, 13,740 ± 600 BP (Pta-116; 14,979-18,568 cal BP), is based on the analysis of bone collagen from the femur of JS 43 in 1988 (36). Due to the poor collagen preservation at the site, the original date has been challenged (66) or ignored (67-68) by some; and an additional nine dates were recently performed using bone, enamel and dentine bioapatite on four other individuals (JS 15, JS 22, JS 42 and JS 103; 65). The bioapatite results ranged from 7,251 to 11,660 BP, with the dates derived from the enamel being systematically younger (7,251-9,687 BP) than the ones obtained from bone and dentine apatite of the same individuals (10,032-11,660 BP; **Table S1**). There is a higher risk of contamination when dating the mineral fraction of bones and teeth due to possible isotopic exchanges between carbonate in bioapatite and dissolved inorganic carbon from the environment during fossilization, especially when a precipitation of secondary carbonates

occurs (69-70). In Jebel Sahaba, this phenomenon is well documented, with a calcified crust deposits over the grave pits, as well as on some skeletal remains (60). Contamination usually results in the dates being too young (69). Consequently, the dentine date (UBA-20132 11,660 BP, 13,362-13,727 cal BP) provides the best apatite age estimate for the site and indirectly confirms the validity of the bone collagen date performed in the eighties (65). Broadly dated between 13,400 and 18,600 cal BP, the Jebel Sahaba cemetery is the earliest funerary complex from the Nile Valley.

SI Text S3: Description of the lithic assemblage

According to Wendorf and Schild (36), 116 lithic pieces were found in direct association with 24 individuals. This number, however, differs from the number of listed artefact in their tables (n=112), as well as those described in the original publication (n=118; 60). Their 2004 paper does not appear to include burials JS C-1, JS C-2 and JS C-3, excavated by the Columbia Expedition and the lithic artefact found with JS 41 was not reported in Wendorf and Schild (36) as its exact position in the deposit was noted as unknown in Wendorf (60).

Our reassessment of the Jebel Sahaba lithic collection housed in the British Museum includes the lithic assemblage from the surface (n=72), and 115 pieces from the original collection described as directly associated to the skeletons. The analysis of the 115 lithic artefacts revealed 62 points, 43 unretouched flakes and microchips, and 7 undetermined pieces (**Fig. S5a, b**). In addition, one Levallois core (**Fig. S5a:c**), one scraper (**Fig. S5a:d**) and one burin (**Fig. S5a:e**) were noted. Among the points, three main morphologies can be distinguished: backed asymmetrical mono-points with an oblique or transverse distal cutting edge (n=22; **Fig. S5a:q-v**), backed symmetrical mono-points (n=16; **Fig. S5a:f-l**) and crescent-like backed pieces (n=9; **Fig. S5a:m-p**). In addition, there are indeterminate points (n=10; mostly fragments which could belong to one of the previous categories). There are also unretouched symmetrical mono-points (n=5; **Fig. S5a:a-b**), some of which are the only ones that can be tentatively assigned to Levallois industry. We also noted the absence of elongated bi-points corresponding to "typical" lunate. The range in size is fairly diverse, with most points microlithic (around 2-2.5 cm long), while others are more robust (3 to 4 cm long). This is especially the case among the backed symmetrical mono-points and the unretouched symmetrical mono-points.

Since its first description, the lithic assemblage associated to the Jebel Sahaba skeletons was described as showing close resemblance with the Qadan industry (60). The latter being characterized as a microlithic industry, mostly microlithic flakes but also bladelets in some context, obtained by non-Levallois single and opposed plateform cores (but Levallois production is noted in some context). It is also characterized by many types of tools such as lunate, endscrapers, sidescapers, backed pieces, burins and points. Bone tools are rare, but grinding stones are common notably in Tushka 8905 (16, 22, 25, 34, 60, 71). All the different loci belonging to Tushka 8905 archaeological site are situated on the left bank of the river Nile, north of Jebel Sahaba (**Fig. S1**) and are, together, considered as a reference site for the Qadan industry (16, 34).

The comparison we were able to make between the artefacts found in direct association of the Jebel Sahaba burials and the assemblage of Tushka 8905, especially the industry from locus B of this site, allows us to maintain this cultural association. The Jebel Sahaba lithic artefacts were mainly produced using a bipolar technique on anvil with small flint nodules or small pebbles. This kind of knapping generates flakes with very diverse morphologies, which explains their high diversity from a typological point of view. For instance, of the 21 pieces found in association to burial JS 44, six are micro- and three unretouched flakes, five are backed symmetrical mono-points, three backed mono-points with an oblique or transverse distal cutting edge, three crescent-like backed pieces and one is an indeterminate point. However, certain intentions are discernable, particularly a wish to obtain elongated flakes with asymmetrical sections. Another method, although less represented, enters into Levallois production *sensu lato*. On the other hand, no blades or bladelets productions are identified in this assemblage.

While acknowledging the specific function of the Jebel Sahaba lithic assemblage, its similarity with Tuskha 8905 stands, particularly our comparison with the industry found in Tuskha's locus B. In both cases, it is an industry dominated by the exploitation of small pebbles or small flint nodules, with a bipolar technique using an anvil which generates fragments of various morphologies without little predetermination bar certain intentions (such as elongated flakes with *débordant* edge). Some of these flakes were transformed into sidescrapers and endscrapers at Tushka 8905-B, but the largest proportion of processed products falls into the family of backed pieces, covering the typological range previously described at Jebel Sahaba. Another technological relationship is based on a significant absence: that of bladelet productions. Ultimately, the lithics from Jebel Shaba only differ from those found at Tuskha 8905-B in that they also include a few examples that may come from a Levallois method. However, in our opinion, the differences noted between the lithic assemblages at these two sites lie mostly in their respective functional status. From a typological point of view, the domestic function of Tushka 8905-B is accompanied by tools such as sidescrapers and endscrapers that are practically absent in Jebel Sahaba. In contrast, the typological diversity of points, as well as of other elements used in the manufacture of weapons, is appreciably richer at Jebel Sahaba than at Tushka. This can, once more, be easily be explained by contextual factors. Therefore, our analysis shows that the occupants of the Tushka 8905-B settlement and the wielders of the weapons that were used against the people buried at Jebel Sahaba do indeed belong to the same technological tradition called Qadan.

A remarks should be made on the artefacts found into the fill surrounding the burials. In a recent paper, Usai (34) uses their existence as a basis for questioning the association of all the artifacts discovered at Jebel Sahaba with the burials. Her hypothesis is that the excavation of the burials in an older archaeological levels would have fortuitously mixed all these artefacts with the contents of the tombs. Against this opinion, militate two registers of facts: 1) the clear spatial correlation that exists between many of the lithic remains found in direct association with bodies and the numerous traces of impact that most skeletal remains bear; 2) the fact that the remains found in direct association with the bodies form an assemblage that is completely different from the one collected in the fill surrounding the burials. Of those found in the fill, the rocks used to make a large proportion of the pieces differ from those used to fashion the lithics explicitly associated with the skeletons. These pieces mostly comprise of silicified wood, quartz or quartzite (29 out of 71 pieces studied). Moreover, a careful examination shows that only about 10% of these elements are similar with the artefacts explicitly associated with the skeletons, underlining that these assemblages are of different origins. Finally, and contrary to what Usai (34) asserts, the number of pieces associated with the bodies that are not compatible with weapons is very small: a scraper (JS 29), a Levallois core (JS 41), and a burin (JS 110). Besides, the core is noted as "found in fill adjacent to skeleton, exact position unknown" (60, p. 977) and the burin is described as "found with or near burials" (60, p. 988). This leads us to conclude that there is no, or very little artefact that could be seen as grave goods, but that most if not all the artefacts found in direct association with the skeletons do indeed belong to the weapons that wounded them.

SI Text S4: Projectile Impact Marks (PIMs) terminology

Although embedded lithic or bone artefact fragments are the most direct diagnostic features used to identify projectile impact marks, a growing number of studies are now available to support the classification and interpretation of cutmarks and other puncture or perforation wounds (72-74).

Projectile Impacts Marks (PIMs) were characterized using projectile bone damage identification criteria derived from experimental archaeological research (72-77). The terminology and classification used in this study are characterized by the level of hard tissue projectile penetration defined by O'Driscoll & Thompson (73). The term **drag** denotes cut-like marks with internal parallel longitudinal microstriations at the bottom of the groove and on its borders (**Fig. S6-a,b**). They are characterized by straight and continuous trajectories similar to slicing cutmarks (74). However, they differ from the latter in that they are deeper, with a wide and flat groove floor, and an abrupt angle between its floor and lateral borders (74). They also display a range of specific secondary traits such as cracking, flaking, scraping and bisecting marks (73-74). Bisecting marks are related to bouncing and the movement of the projectile when it comes into contact with bone (73). The shoulder effects found in slicing cutmarks are less pronounced in PIMs, most probably due to the rapidity and singularity of the impact (74, 78-79). Finally, the anatomical location of the PIMs can also be used to differentiate them from slicing cutmarks (73, 75). When the cause of the cut could not be ascertained, the generic term of cutmark is used (80). A mark related to the embedment of a projectile in a bone is define as a **puncture** by O'Driscoll & Thompson (73) and this type of impact can be associated with the crushing, beveling, flaking and splitting of bone (**Fig. S6-c,d**). When the projectile fully penetrates the bone, the term **perforation** is favored (77).

Supplementary Figures

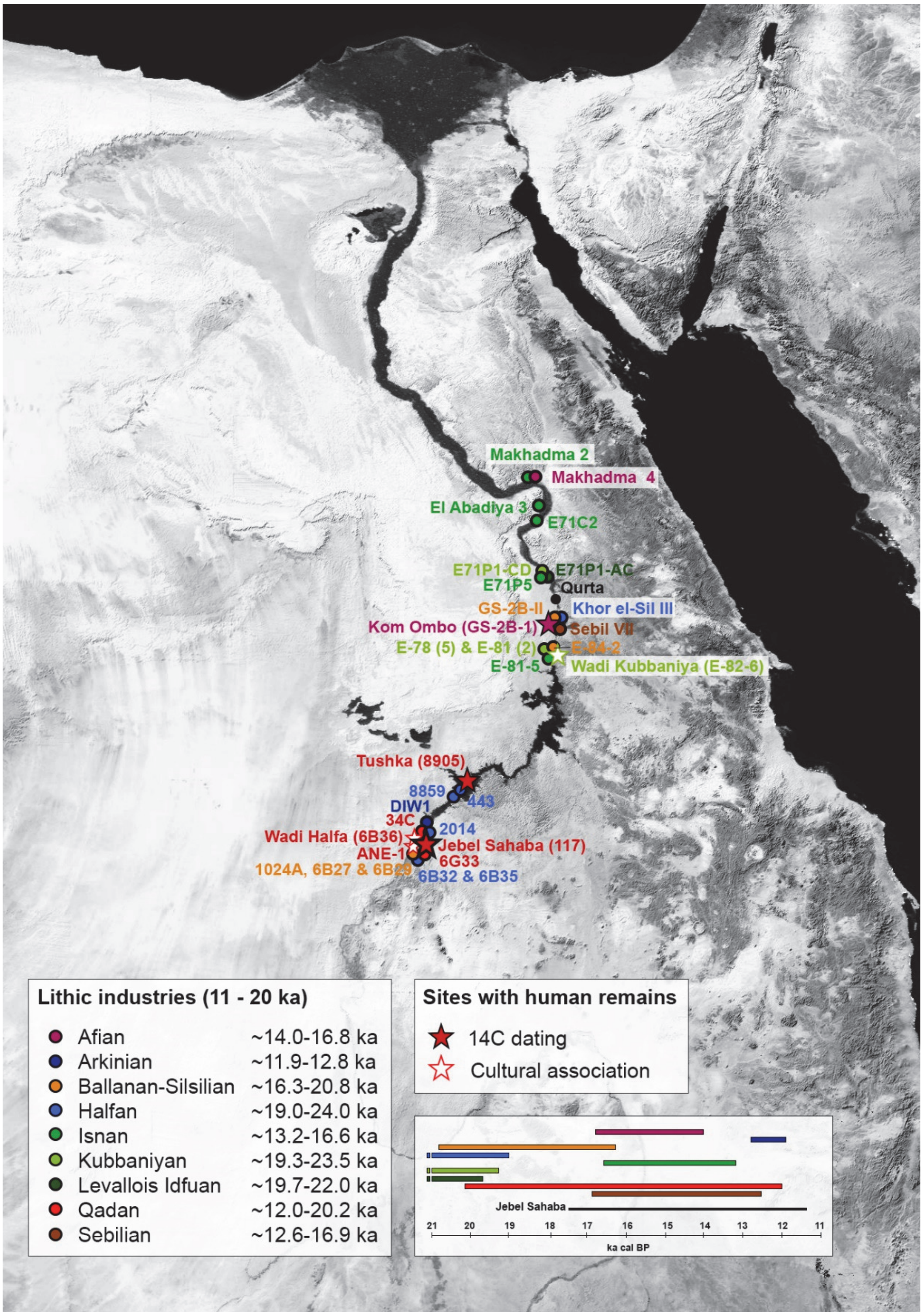

Fig. S1: Map of the archaeological sites with radiocarbon dates and clear cultural association in the Nile Valley between 11-20 ka. Based on Leplongeon's review database (16).

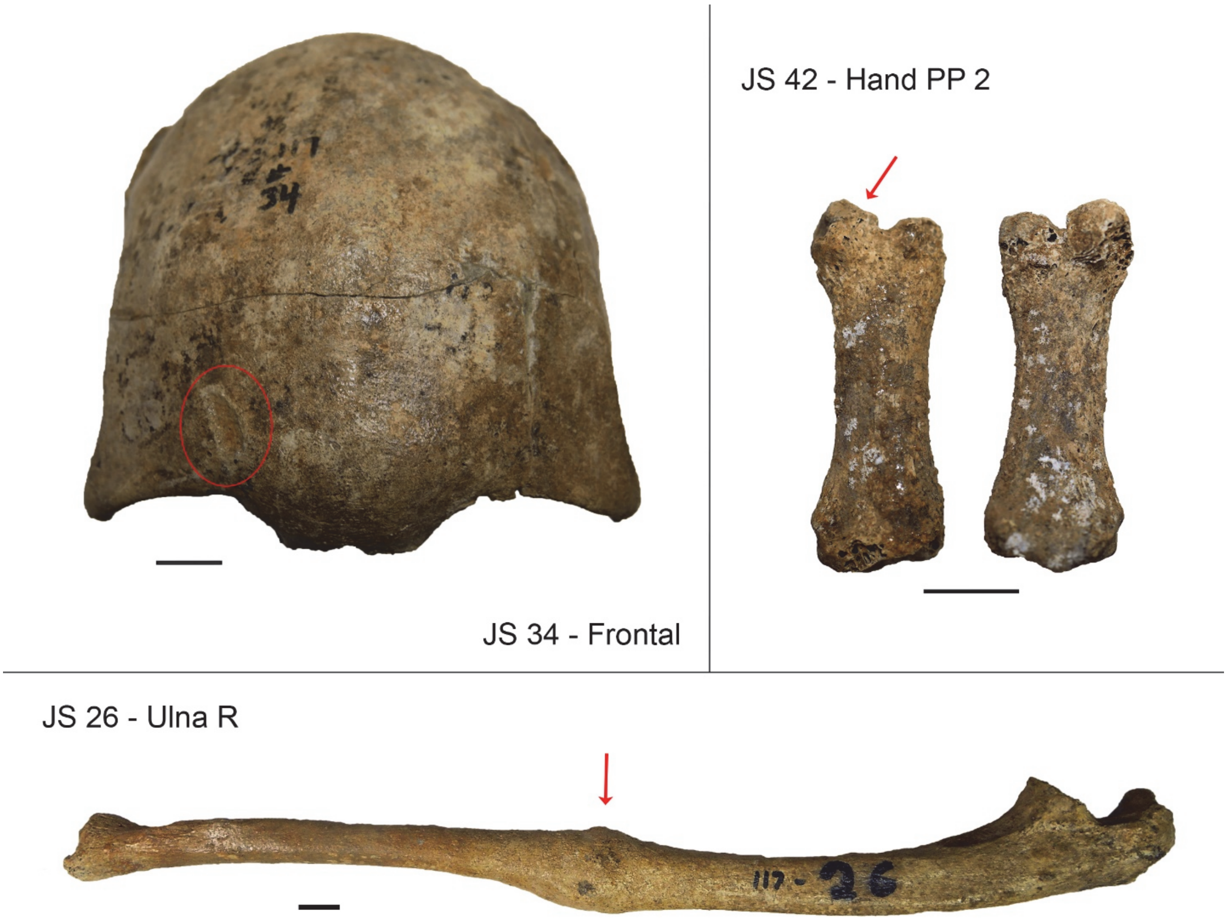

Fig. S2: Illustration of some of the healed lesions recorded on the Jebel Sahaba individuals. Upper left quadrant: ovoid healed injury on the frontal bone; upper right quadrant: healed fracture of the distal extremity of the hand's second proximal phalanx. bottom: healed parry fracture of the ulna. Black bar = 1 cm.

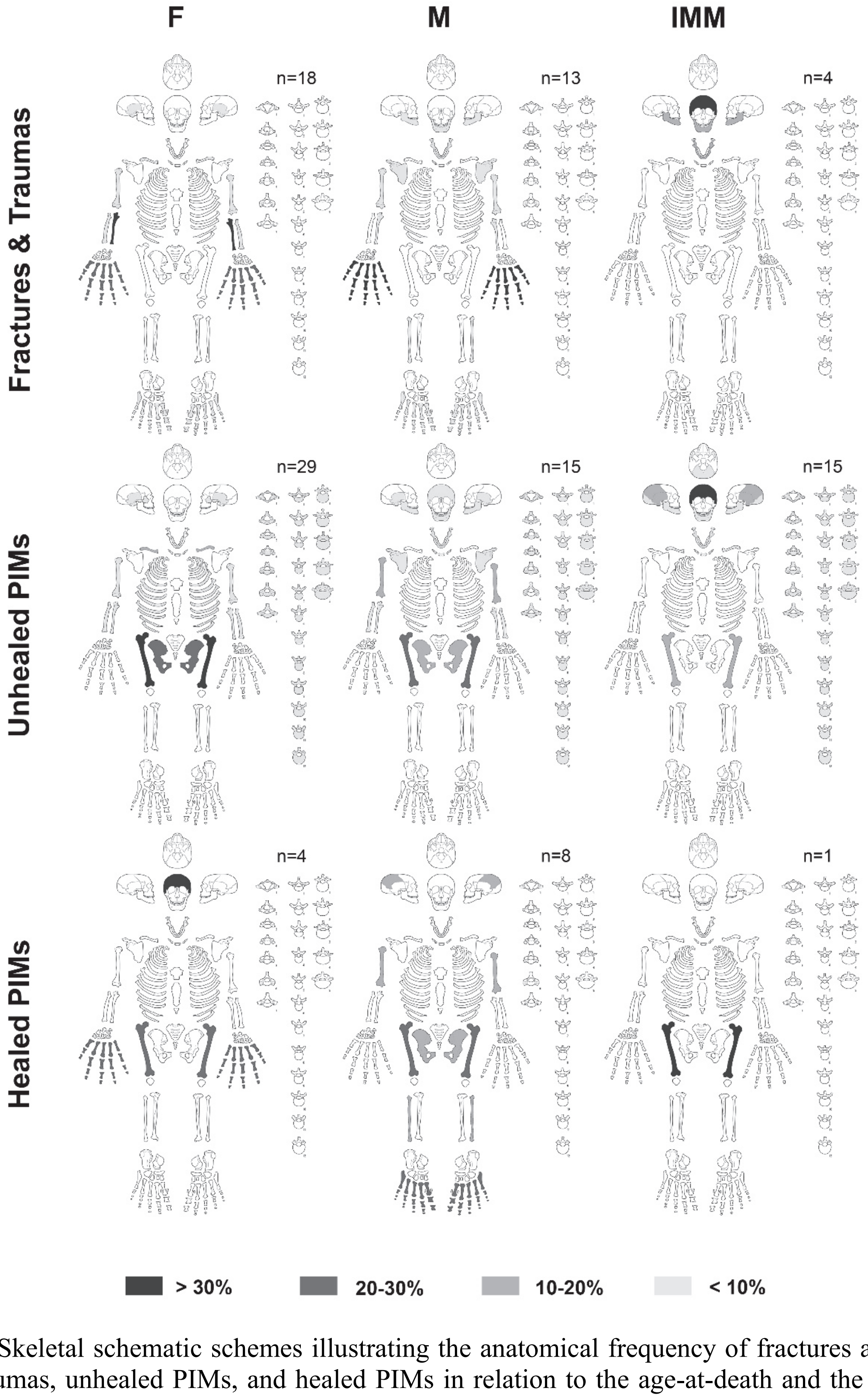

Fig. S3: Skeletal schematic schemes illustrating the anatomical frequency of fractures and blunt force traumas, unhealed PIMs, and healed PIMs in relation to the age-at-death and the sex. F = Female, M = Male, IMM = Immature, n = total number of lesions recorded for each category.

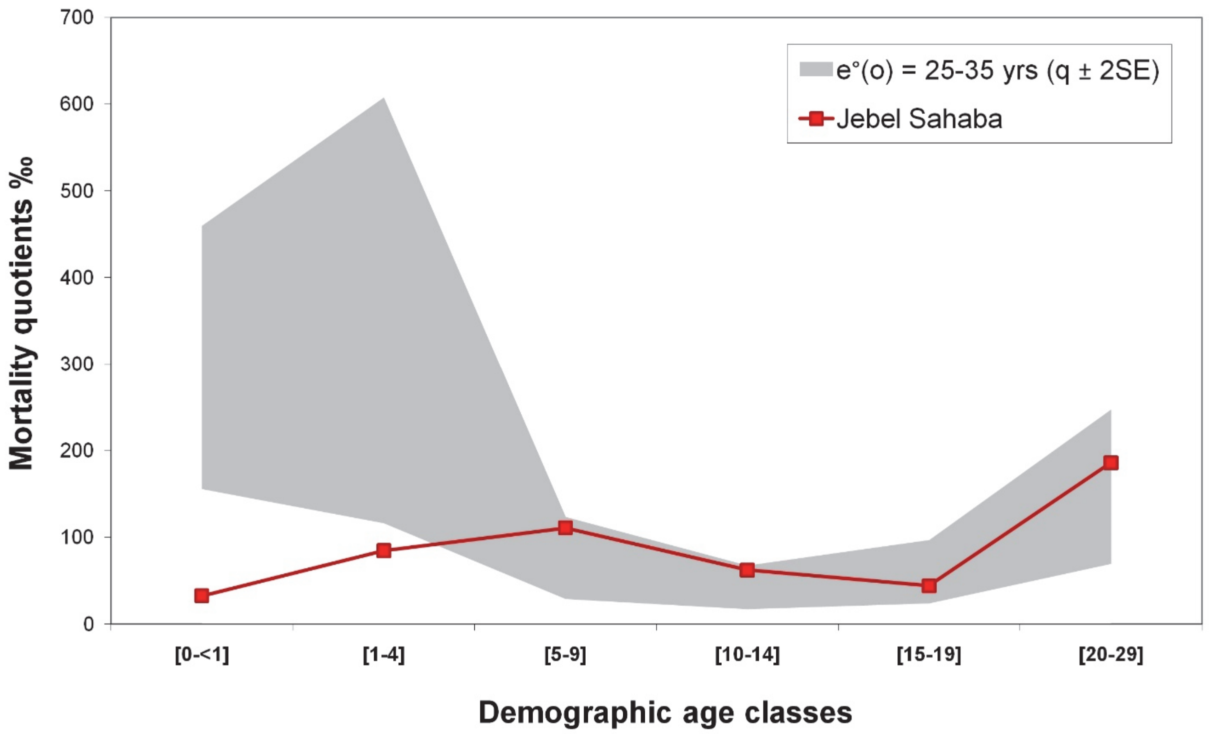

Fig. S4: Mortality quotients by age class at Jebel Sahaba compared to the theoretical mortality rates of Ledermann (81) for a population with a life expectancy at birth of between 25 and 35 years.

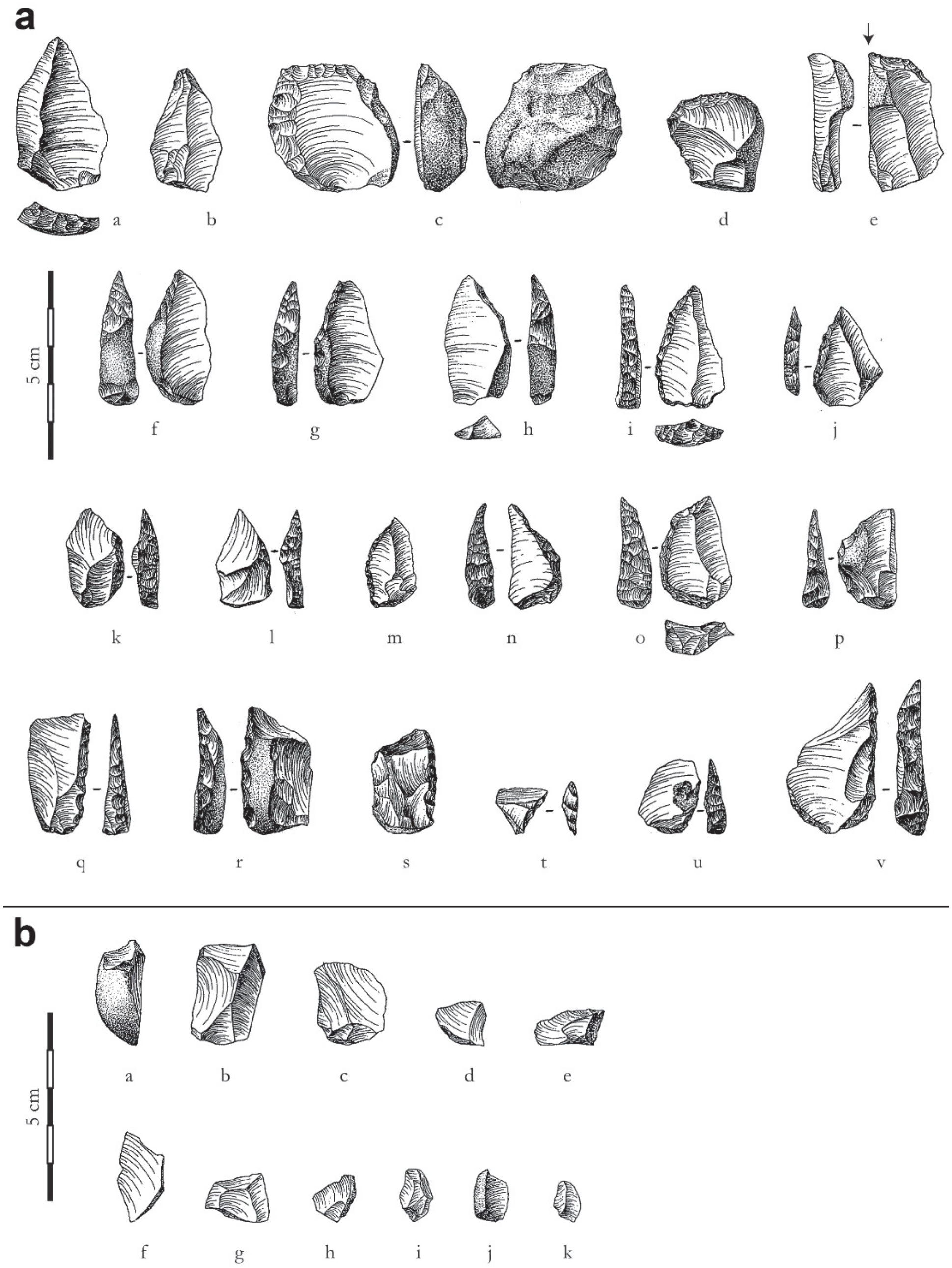

Fig. S5: Jebel Sahaba lithic industry sample.
5a: a-b = unretouched symmetrical mono-points (assimilated with caution to Levallois points), c = Levallois core, d = scraper, e = burin, f-l = backed symmetrical mono-points, m-p = crescent-like backed pieces, q-v = backed asymmetrical mono-points with an oblique or transverse distal cutting edge. Burials JS 14 (g-h), JS 21 (i, m, v), JS 29 (d), JS 31 (q-r), JS 33 (s), JS 34 (n), JS 35 (a, t), JS 41 (c), JS 44 (f, j-k-l, p, u), JS 103 (b), with or near JS 110 (e), JS-C1-3 (o) (modified following 60). 5b: unretouched flakes and microflakes (complete or fragmented) samples from Burials JS 21 (c), JS 23 (e), JS 29 (a), JS 31 (b), JS 33(d), JS 44 (f-k) (modified following 60).

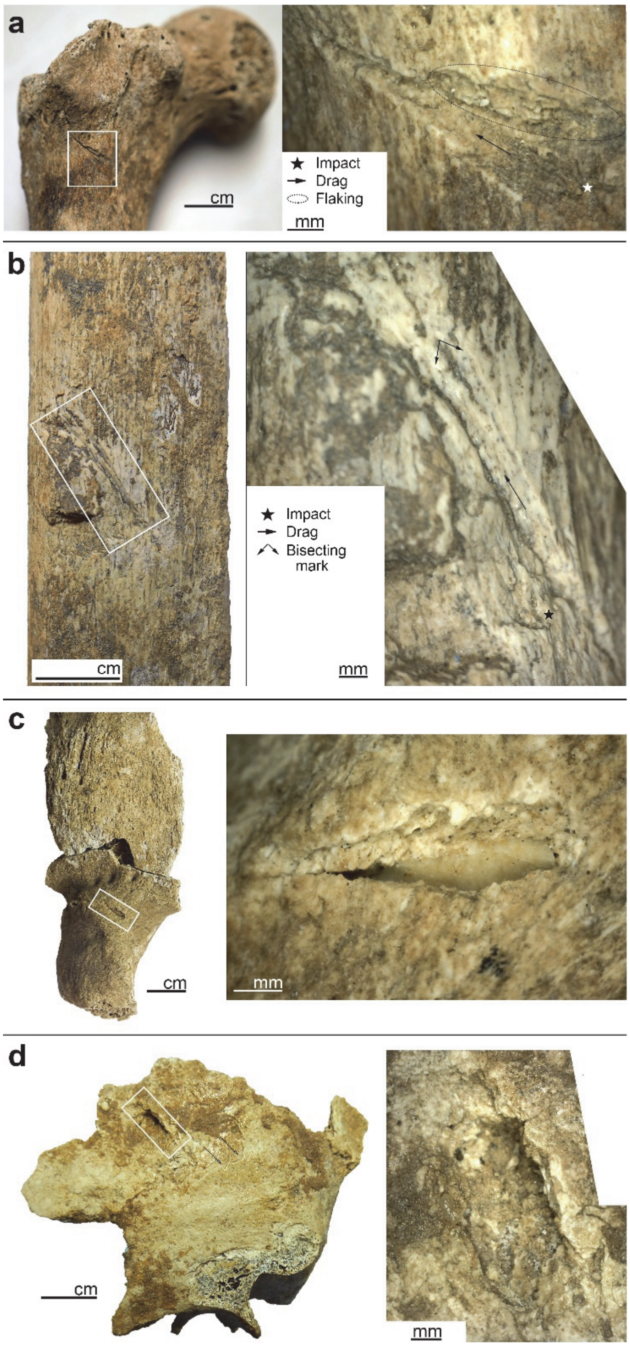

Fig. S6: Illustration drag and puncture types of Projectile Impact Marks (PIM) seen on the Jebel Sahaba individuals. a: Macroscopic (left) and microscopic (right) views of a drag with bone flaking on the JS 37 right femur. b: Macroscopic view (left) and composite microscopic image (right) of a drag with bisecting marks on the JS 20 left humerus. c: Macroscopic (left) and microscopic (right) views of a puncture with embedded artefact on the JS 21 left coxal. d: Macroscopic view (left) and composite microscopic image (right) of a puncture with crushing fractures associated to the extraction of the projectile on the JS 48 right temporal. The white rectangles in the macroscopic view delimit the magnified area illustrated in the microscopic image.

Supplementary Tables

Table S1. Results of the direct radiocarbon dates of the Jebel Sahaba individuals. Calibration software = Oxcal version 4.4.2 (82). Calibration curve = IntCal 2020 (83).

**Prep #**	**sample #**	**sample ID**	**anatomical part**	**fraction dated**	**^{14}C age**	**error**	**target #**	**Calibrated range (cal BP, 95.4%)**		**Ref.**
Muse103	DS-1	skeleton 15	lower right M3	enamel apatite	7251	31	UBA-20124	8170	7981	65
Muse111	DS-9	skeleton 15	lower right M3	dentine+root apatite	11660	52	UBA-20132	13727	13362	65
Muse110	DS-2	skeleton 15	long bone fgmt	bone apatite	11049	43	UBA-20125	13090	12843	65
Muse104	DS-3	skeleton 22	upper left M3	enamel apatite	8512	40	UBA-20126	9544	9467	65
Muse99	DS-4	skeleton 22	pelvis fgmt	bone apatite	11133	50	UBA-20127	13160	12911	65
Muse105	DS-5	skeleton 42	lower right M3	enamel apatite	9043	45	UBA-20128	10285	9967	65
Muse100	DS-6	skeleton 42	pelvis fgmt	bone apatite	11093	49	UBA-20129	13104	12850	65
-	-	skeleton 43	-	bone collagen	13740	600	Pta-116	18568	14979	36
Muse102	DS-7	skeleton 103	upper right M2	enamel apatite	9687	55	UBA-20130	11229	10792	65
Muse101	DS-8	skeleton 103	pelvis fgmt	bone apatite	10032	46	UBA-20131	11802	11319	65

Table S2: Inventory of Jebel Sahaba individuals showing their biological identity (sexual diagnosis and age-at-death estimates) and the type of lesions recorded for each of them. Lesions are separated in two main columns for healed and unhealed injuries. Each column is subdivided by type of lesions: Lesions (when the origin is unknown); Traumas (for bone fracture, blunt force traumas and perforations whose projectile origin is not ascertained) and PIMs (for Projectile Impact Marks). The presence of embedded lithic artefacts in association to PIMs is also listed. UND = undeterminate; M = Male; pM = probable; pM? = possible Male; F = Female, pF = probable Female; pF? = possible Female; MA = Mature; IMM = Immature; L = Lesion; T = Trauma; PIMs = Projectile Impact Marks; EmL = Embedded lithics; *Sexual diagnosis and age-at-death estimates differing from Wendorf (60).

Individual	Biological identity				Healed				Unhealed			
	Sex	Mature/ Immature	Age-at-death estimates	Demographic cohorts	L	T	PIMs	EmL	L	T	PIMs	EmL
JS 2	UND	IMM	[5-9]/[10-14]	[5-9]		x						
JS 4	UND*	IMM	[15-19]/[20-30]*	[15-19]	x							
JS 5	pM	MA	[20-49]	[> 30]	x	x			x			
JS 6	M	MA	[> 40]	[> 30]	x		x	x			x	
JS 7	pF?	MA	[> 30]	[> 30]		x						
JS 8	pF?	MA	[> 30]	[> 30]		x	x					
JS 9	UND	IMM	[1-4]/[5-9]	[1-4]							x	
JS 10	pM?	MA	[25-39]	[> 30]								
JS 11A	pM?	MA	[20-35]	[20-29]		x						
JS 11B	UND	MA	[> 30]*	[> 30]	x							
JS 11C	UND	IMM	[0]/[0<1]*	[0-<1]								
JS 12	UND	IMM	[5-9]	[5-9]								
JS 13	UND	IMM	[1-4]/[5-9]*	[1-4]								
JS 14	UND	IMM	[1-4]*	[1-4]						x	x	x
JS 15	pF?	MA	[20-35]	[20-29]	x		x					
JS 16	pF?	MA	[> 30]	[> 30]			x				x	
JS 17	pM?	MA	[20-35]	[20-29]								
JS 18	pM	MA	[> 50]	[> 30]								
JS 19	M	MA	[> 30]	[> 30]	x	x						
JS 20	pM?	MA	[> 30]	[> 30]		x			x		x	x
JS 21	pF*	MA	[30-49]	[> 30]	x						x	x
JS 22	M*	MA	[25-39]	[> 30]			x	x				
JS 23	pF?	MA	[> 30]	[> 30]	x						x	x
JS 24	UND	IMM	[5-9]*	[5-9]							x	
JS 25	pF?*	MA	[> 50]	[> 30]								
JS 26	F	MA	[20-49]	[> 30]		x					x	
JS 27	UND	IMM	[0-1]/[1-4]*	[1-4]								
JS 28	pF	MA	[20-49]	[> 30]								
JS 29	M	MA	[20-49]	[> 30]		x					x	x

Individual	Biological identity				Healed				Unhealed			
	**Sex**	**Mature/ Immature**	**Age-at-death estimates**	**Demographic cohorts**	**L**	**T**	**PIMs**	**EmL**	**L**	**T**	**PIMs**	**EmL**
JS 31	pM	MA	[> 30]	[> 30]	x	x	x	x	x		x	x
JS 32	UND	MA	[25-35]*	[20-29]		x	x					
JS 32b	UND	MA	[> 30]	[> 30]		x						
JS 33	pF?	MA	[25-35]	[20-29]		x					x	
JS 34	pF?	MA	[20-49]*	[> 30]	x	x						
JS 35	UND	MA	[20-35]	[20-29]	x							
JS 36	pF?	MA	[> 30]*	[> 30]								
JS 37	pF?	MA	[> 30]	[> 30]	x						x	
JS 37b	M	MA	[> 30]	[> 30]								
JS 38	M	MA	[> 30]	[> 30]	x	x	x					
JS 39	pM	MA	[> 30]	[> 30]	x	x						
JS 40	pM	MA	[15-19]/[20-25]	[20-29]	x	x						
JS 41	pF*	MA	[> 30]*	[> 30]	x	x			x			
JS 42	pM	MA	[20-29]	[20-29]	x	x					x	
JS 43	UND*	MA	[> 50]	[> 30]	x	x						
JS 44	pF?	MA	[> 30]*	[> 30]		x					x	x
JS 45	pF?	MA	[> 30]	[> 30]								
JS 46	pM*	MA	[> 30]	[> 30]								
JS 47	UND	IMM	[5-9]	[5-9]								
JS 48	pM	IMM	[15-19]*	[15-19]		x					x	
JS 49	pF	MA	[> 30]	[> 30]								
JS C1	M	MA	[>50]	[> 30]			x	x	x			
JS C2	UND	IMM	[1-4]/[5-9]	[5-9]								
JS C3	UND	MA	[> 30]	[> 30]								
JS 100	UND	IMM	[1-4]/[5-9]	[5-9]								
JS 100 extra	UND	IMM	[0-<1]*	[0-<1]								
JS 101A-B	UND	IMM	[1-4]*	[1-4]								
JS 102	pF	MA	[> 30]	[> 30]	x	x	x					
JS 103	UND*	IMM	[10-14]*	[10-14]							x	x
JS 104-107	pF?	MA	[> 30]	[> 30]	x	x						
JS 105	UND	IMM	[10-14]/[15-19]	[10-14]	x	x						
JS 106	M	IMM	[10-14]/[15-19]*	[10-14]			x					

Table S3: Inventory of Jebel Sahaba individuals with the burial characteristics and associated artefacts. UND = undeterminate; M = Male; pM = probable; pM? = possible Male; F = Female, pF = probable Female; pF? = possible Female. #artefact absent from the British Museum collection. *additional artefact compared to the original publication.

**Individual**	**Sex**	**Demographic age class**	**Presence of lesions**	**Burial**	**Grave pit**		**Artefact associated** (Wendorf, 1968)		**New embedded artefacts** (this study)
					**Depth**	**Cover**	**volume of the body**	**embedded**	
JS 2	UND	[5-9]	x	Disturbed	At surface				
JS 4	UND	[15-19]	x	Disturbed	15 cm	Slabs			
JS 5	pM	[> 30]	x	Individual	10 cm	Slabs			
JS 6	M	[> 30]	x	Disturbed	10 cm	Slabs			1
JS 7	pF?	[> 30]	x	Individual	25 cm				
JS 8	pF?	[> 30]	x	Double	30 cm				
JS 9	UND	[1-4]	x	Double	30 cm				
JS 10	pM?	[> 30]		Individual	30 cm				
JS 11A	pM?	[20-29]	x	Disturbed	10 cm				
JS 11B	UND	[> 30]	x	Disturbed	10 cm				
JS 11C	UND	[0-<1]		Disturbed	10 cm				
JS 12	UND	[5-9]		Individual	10 cm				
JS 13	UND	[1-4]		Double	25 cm		2		
JS 14	UND	[1-4]	x	Double	25 cm		3		1
JS 15	pF?	[20-29]	x	Individual	35 cm				
JS 16	pF?	[> 30]	x	Individual	35 cm				
JS 17	pM?	[20-29]		Individual	35 cm		1		
JS 18	pM	[> 30]		Individual	30 cm	Slabs			
JS 19	M	[> 30]	x	Individual	40 cm	Slabs			
JS 20	pM?	[> 30]	x	Double	40 cm	Slabs	6		1
JS 21	pF	[> 30]	x	Double	40 cm	Slabs	17	2#	6
JS 22	M	[> 30]	x	Individual	40 cm	Slabs			1
JS 23	pF?	[> 30]	x	Double	10-20 cm		2	1#	
JS 24	UND	[5-9]	x	Double	40 cm		1		
JS 25	pF?	[> 30]		Multiple	45 cm	Slabs	1#		
JS 26	F	[> 30]	x	Multiple	30 cm	Slabs	5 (+1*)		
JS 27	UND	[1-4]		Disturbed	20 cm	On slabs			
JS 28	pF	[> 30]		Multiple	20 cm		1		
JS 29	M	[> 30]	x	Multiple	40 cm	Slabs	7		
JS 31	pM	[> 30]	x	Individual	30 cm	Slabs	15	2#	1
JS 32	UND	[20-29]	x	Disturbed	60 cm				
JS 32b	UND	[> 30]	x	Disturbed					
JS 33	pF?	[20-29]	x	Individual	60 cm	Slabs	8		

Individual	Sex	Demographic age class	Presence of lesions	Burial	Grave pit		Artefact associated (Wendorf, 1968)		New embedded artefacts (this study)
					Depth	Cover	volume of the body	embedded	
JS 34	pF?	[> 30]	x	Multiple	60 cm	Slabs	2		
JS 35	UND	[20-29]	x	Disturbed	50 cm	Slabs	6		
JS 36	pF?	[> 30]		Individual	40 cm	Slabs			
JS 37	pF?	[> 30]	x	Multiple	60 cm	Slabs	1		
JS 37b	M	[> 30]		Multiple					
JS 38	M	[> 30]	x	Individual	60 cm	Slabs	1		
JS 39	pM	[> 30]	x	Individual	70 cm	Slabs			
JS 40	pM	[20-29]	x	Individual	70 cm	Slabs			
JS 41	pF	[> 30]	x	Individual	40 cm	Slabs	1		
JS 42	pM	[20-29]	x	Individual	35 cm	Slabs	1		
JS 43	UND	[> 30]	x	Individual	35 cm	Slabs			
JS 44	pF?	[> 30]	x	Individual	35 cm	Slabs	20	1	1
JS 45	pF?	[> 30]		Individual	40 cm	Slabs	1[#]		
JS 46	pM	[> 30]		Disturbed	40 cm	Slabs			
JS 47	UND	[5-9]		Individual	35 cm	On slabs	1[#]		
JS 48	pM	[15-19]	x	Individual	50 cm				
JS 49	pF	[> 30]		Individual	45 cm	Slabs			
JS C1	M	[> 30]	x	Multiple	At surface	Slabs			1
JS C2	UND	[5-9]		Multiple	At surface	Slabs	5		
JS C3	UND	[> 30]		Multiple	At surface	Slabs			
JS 100	UND	[5-9]		Multiple	70 cm	Slabs			
JS 100 extra	UND	[0-<1]		Multiple					
JS 101A-B	UND	[1-4]		Multiple	70 cm	Slabs			
JS 102	pF	[> 30]	x	Multiple	70 cm	Slabs	1		
JS 103	UND	[10-14]	x	Multiple	65 cm	Slabs	1	1[#]	
JS 104 & JS 107	pF?	[> 30]	x	Disturbed	70-90 cm				
JS 105	UND	[10-14]	x	Individual	90 cm				
JS 106	M	[10-14]	x	Individual	60 cm	Slabs	1		

Supplementary references

(1) Gasse, F., 2000. Hydrological changes in the African tropics since the Last Glacial Maximum. *Quaternary Science Reviews* 19: 189-211.

(2) deMenocal, P., Ortiz, J., Guilderson, T., Adkins, J., Sarnthein, M., Baker, L. & Yarusinsky, M., 2000. Abrupt onset and termination of the African Humid Period: rapid climate responses to gradual insolation forcing. *Quaternary Science Reviews* 19: 347-361.

(3) Mayewski, P.A., Rohling, E.E., Stager, J.C., Karlén, W., Maasch, K.A., Meeker, L.D., Meyerson, E.A., Gasse, F., van Kreveld, S., Holmgren, K., *et al.*, 2004. Holocene climate variability. *Quaternary Research* 62: 243-255.

(4) Nicoll, K., 2004. Recent environmental change and prehistoric human activity in Egypt and Northern Sudan. *Quaternary Science Reviews* 23 (5-6): 561-580.

(5) Kuper, R. & Kröpelin, S., 2006. Climate-controlled Holocene occupation in the Sahara: motor of Africa's evolution. *Science* 313: 803-807.

(6) Williams, M., Talbot, M., Aharon, P., Abdl Salaam, Y., Williams, F., & Brendeland K.I., 2006. Abrupt return of the summer monsoon 15,000 years ago: new supporting evidence from the lower White Nile valley and Lake Albert. *Quaternary Science Reviews* 25: 2651-2665.

(7) Williams, M.A.J., Williams, F.M., Duller, G.A.T., Munro, R.N., El Tom, O.A.M., Barrows, T.T., Macklin, M., Woodward, J., Talbot, M.R., Haberlah, D. & Fluin, J., 2010. Late Quaternary floods and droughts in the Nile valley, Sudan: new evidence from optically stimulated luminescence and AMS radiocarbon dating. *Quaternary Science Reviews* 29: 1116-1137.

(8) Honegger, M. & Williams, M., 2015. Human occupations and environmental changes in the Nile valley during the Holocene: The case of Kerma in Upper Nubia (northern Sudan). *Quaternary Science Reviews* 130: 141-154.

(9) Macklin, M.G., Toonen, W.H.J., Woodward, J.C., Williams, M.A.J., Flaux, C., Marriner, N., Nicoll, K., Verstraeten, G., Spencer, N., & Wesby, D., 2015. A new model of river dynamics, hydroclimatic change and human settlement in the Nile Valley derived from meta-analysis of the Holocene fluvial archive. *Quaternary Science Reviews* 130: 109-123.

(10) Vermeersch, P.M. & Van Neer, W., 2015. Nile behaviour and Late Palaeolithic humans in Upper Egypt during the Late Pleistocene. *Quaternary Science Reviews* 130: 155-167.

(11) Williams, M.A.J., Usai, D., Salvatori, S., Williams, F.M., Zerboni, A., Maritan, L. & Linseele, V., 2015. Late Quaternary environments and prehistoric occupation in the lower White Nile valley, central Sudan. *Quaternary Science Reviews* 130: 72-88.

(12) Castañeda, I. S., Schouten, S., Pätzold, J., Lucassen, F., Kasemann, S., Kuhlmann, H. & Schefuß, E., 2016. Hydroclimate variability in the Nile River Basin during the past 28,000 years. *Earth and Planetary Science Letters* 438: 47-56.

(13) Sanchez Goñi, M.F. & Harrison, S.P., 2010. Millennial-scale climate variability and vegetation changes during the Last Glacial: Concepts and terminology. *Quaternary Science Reviews* 29: 2823-2827.

(14) Hoelzmann, P., Gasse, F., Dupont, L.M., Salzmann, U., Staubwasser, M., Leuschner, D.C., Sirocko, F., 2004. 12. Palaeoenvironmental changes in the arid and subarid belt (Sahara-Sahel-Arabian peninsula) from 150 kyr to present. In: Battarbee, R.W., Gasse, F. & Stickley, C.E.

(Eds), Past Climate Variability through Europe and Africa. Volume 6. Springer, Dordrecht, pp. 219-256.
(15) Vermeersch, P.M., 2020. Human occupation density and mobility in the lower Nile Valley. *In*: Leplongeon, A., Goder-Goldberger, M. & Pleurdeau, D. (Eds), *Not Just a Corridor. Human occupation of the Nile Valley and neighbouring regions between 75,000 and 15,000 years ago*. MNHN, Paris, pp. 139-157.
(16) Leplongeon, A., 2021. The main Nile Valley at the End of the Pleistocene (28-15ka): Dispersal Corridor or Environmental Refugium ? *Frontiers in Earth Science* 8: 607183.
(17) Williams, M.A.J., Adamson, D., Cock, B., McEvedy, R., 2000. Late Quaternary environments in the White Nile region, Sudan. Global and Planetary Change 26: 305-316.
(18) Paulissen, E. & Vermeersch, P.M., 1987. Earth, man and climate in the Egyptian Nile Valley during the Pleistocene. *In*: Close, A. (ed.), *Prehistory of Arid North Africa*. SMU Press, Dallas, pp. 29-67.
(19) Rasmussen, S.O., Andersen, K.K., Svensson, A.M., Steffensen, J.P., Vinther, B.M., Clausen, H.B., Siggaard-Andersen, M.-L, Johnsen, S.J., Larsen, L.B., Dahl-Jensen, D., *et al.*, 2006. A new Greenland ice core chronology for the last glacial termination. *Journal of Geophysical Research* 111: D06102.
(20) Butzer, K.W., 1980. Pleistocene history of the Nile Valley in Egypt and Lower Nubia. *In*: Williams, M.A.J. & Faure, H. (Eds), *The Sahara and the Nile. Quaternary environments and Prehistoric occupations in northern Africa*. Balkema, Rotterdam, 253-280.
(21) Dufour, E., Van Neer, W., Vermeersch, P. & Patterson, W.P., 2018. Hydroclimatic conditions and fishing practices at Late Paleolithic Makhadma 4 (Egypt) inferred from stable isotope analysis of otoliths. Quaternary International 471: 190-202.
(22) Schild, R. & Wendorf, F., 2010. Late Palaeolithic Hunter-Gatheres in the Nile Valley of Nubia and Upper Egypt. *In*: Garcea, E.A.A. (ed.), *South-eastern Mediterranean Peoples Between 130,000 and 10,000 Years Ago*. Oxbow Books, Oxford and Oakville, pp. 89-125.
(23) Osypińska, M., Osypiński, P., Chłodnicki, M., Kuc, M., Wiktorowicz, P. & Ryndziewicz, R., 2020. The PalaeoAffad Project and the Prehistory of the Middle Nile. *Archaeologia Polona* 58: 79-97.
(24) Wendorf, F., 1968. The Late Paleolithic sites in Egyptian Nubia. *In*: Wendorf, F. (ed), *The Prehistory of Nubia, Volume 2*. Fort Burgwin Research Center and Southern Methodist University Press, Dallas, pp. 791-953.
(25) Shiner, J.L., 1968. The Cataract Tradition. *In*: Wendorf, F. (ed), *The Prehistory of Nubia, Volume 2*. Fort Burgwin Research Center and Southern Methodist University Press, Dallas, pp. 535-629.
(26) Wendorf, F., Schild, R. & Close, A.E. (Eds.) 1989. *The Prehistory of Wadi Kubbaniya*. *Volume 2 and 3*. Southern Methodist University, Dallas.
(27) Vermeersch, P.M., 2010. Middle and Upper Palaeolithic in the Egyptian Nile Valley. *In*: Garcea, E.A.A. (ed.), *South-eastern Mediterranean Peoples Between 130,000 and 10,000 Years Ago*. Oxbow Books, Oxford and Oakville, pp. 66-88.
(28) Van Neer, W., Paulissen, E. & Vermeersch, P.M., 2000. Chronology, subsistence and environment at the Late Palaeolithic fishing sites of Mahatma 2 and 4. *In*: Vermeersch, P.M.

(Ed), *Palaeolithic living sites in Upper and Middle Egypt*. Leuven University Press, Leuven, pp. 271-288.
(29) Linseele, V. & Van Neer, W., 2010. Exploitation of desert and other wild game in ancient Egypt: the archaeozoological evidence from the Nile Valley. *In*: Riemer, H., Förster, F., Herb, M., & Pöllath, N. (Eds), *Desert Animals in the Eastern Sahara: Their Position, Significance and Cultural Reflection in Antiquity. Colloquium Africanum 4*. Heinrich Barth Institute, Köln, pp. 47-78.
(30) Yeshurun, R., 2018. Taphonomy of old archaeofaunal collections: New site-formation and subsistence data for the Late Paleolithic Nile Valley. *Quaternary International* 471: 35-54.
(31) Huyge, D., Vandenberghe, D.A.G., De Dapper, M., Mees, F., Cleas, W., Darnell, J.C., 2011. First evidence of Pleistocene rock art in North Africa: securing the age of the Qurta petroglyphs (Egypt) through OSL dating. *Antiquity* 85: 1184-1193.
(32) Vermeersch, P.M. (Ed.), 2000. *Palaeolithic living sites in Upper and Middle Egypt*. Leuven University Press, Leuven, 330 p.
(33) Usai, D., 2008. Lunates and micro-lunates, cores and flakes: the lithic industry of R12. *In*: Salvatori, S. & Usai, D. (Eds), *A neolithic cemetery in the Northern Dongola reach (Sudan): excavation at site R12*. BAR international series, Archaeopress, Oxford, pp. 33-52.
(34) Usai, D., 2020. The Qadan, the Jebel Sahaba Cemetery and the Lithic Collection. *Archaeologia Polona* 58: 99-119.
(35) Leplongeon, A., 2017. Technological variability in the late Palaeolithic lithic industries of the Egyptian Nile Valley: the case of the silsilian and afian industries. *PLoS ONE* 12: e0188824.
(36) Wendorf F. & Schild R., 2004. Late Paleolithic Warfare in Nubia: The Evidence and Causes. *Aduma* 10: 7-28.
(37) Nicoll, K., 2001. Radiocarbon Chronologies for Prehistoric Human Occupation and Hydroclimatic Change in Egypt and Northern Sudan. *Geoarchaeology: An International Journal* 16: 47-64.
(38) Bubenzer, O. & Reimer, H., 2007. Holocene Climatic Change and Human Settlement Between the Central Sahara and the Nile Valley: Archaeological and Geomorphological Results. *Geoarchaeology: An International Journal* 22: 607-620.
(39) Manning, K. & Timpson, A., 2014. The demographic response to Holocene climate change in the Sahara. *Quaternary Science Reviews* 101: 28-35.
(40) Vermeersch, P.M., 2001. "Out of Africa" from an Egyptian point of view. *Quaternary International* 75: 103-112.
(41) Dittrich, A., 2015. Dating the Neolithisation process in the Middle Nile valley: a critical approach. *In*: Kabacinski, J., Chlodnicki, M. & Kobusiewicz, M. (Eds.), *Hunter-Gatherers and Early Food Producing Societies in Northeastern Africa, Studies in African Archaeology*. Poznan, Poznan Archaeological Museum, pp. 15-64.
(42) Clark, J.D., 1989. Shabona: an Early Khartoum settlement on the White Nile. *In*: Krzyzaniak, L. & Kobusiewicz, M. (Eds.), *Late Prehistory of the Nile Basin and the Sahara*. Poznan Archaeological Museum, pp. 417-419.
(43) Usai, D., Salvatori, S., Lacumin, P., Di Matteo, A., Jakob, T. & Zerboni, A., 2010. Excavating a unique pre-Mesolithic cemetery in central Sudan. *Antiquity*. Report (publish on the internet site).

(44) Suková, L. & Varadzin, L., 2012. Preliminary report on the exploration of Jebel Sabaloka (West Bank), 2009-2012. *Sudan & Nubia* 16: 118-131.
(45) Jesse, F., 2010. Early Pottery in Northern Africa - An Overview. *Journal of African Archaeology* 8: 219-238.
(46) Haaland, R., 2015. Origin of Domestication and Aquatic Adaptation: The Nile Valley in Comparative Perspective. *In*: Tvedt, T. & Oestigaard, T. (Eds.), *A History of Water Series, Volume 3*. Water and Food. L.B. Tauris, pp 55-74.
(47) Wendorf, F. & Schild, R., 1998. Nabta Playa and Its Role in Northeastern African Prehistory. *Journal of Anthropological Archaeology* 17: 97-123.
(48) Kuper, R. & Riemer, H., 2013. Herders before pastoralism: prehistoric prelude in the eastern Sahara. *In*: Bollig, M., Schnegg, M. & Wotzka, H.-P. (Eds.), *Pastoralism in Africa. Past Present and Future*. Berghahn. New York/Oxford, pp. 31-65.
(49) Stock, F. & Gifford-Gonzales, D., 2013. Genetics and African Cattle Domestication. *African Archaeological Review* 30: 51-72.
(50) Brass, M., 2018. Early North African Cattle Domestication and Its Ecological Setting: A Reassessment. *Journal of World Prehistory* 31: 81-115.
(51) Wendorf, F. & Schild, R., 1980. *Prehistory of the eastern Sahara*. New York, N.Y. (USA) Academic Press, 414 p.
(52) Gautier, A., 2001. The Early to Late Neolithic Archeofaunas from Nabta and Bir Kiseiba. *In*: Wendorf, F. & Schild, R. (Eds.), *Holocene settlement of the Egyptian Sahara, Volume 1*. Kluwer Academic / Plenum Publishers, pp. 609-635.
(53) Jórdeczka, M., Krolik, H., Masojc, M. & Schild, R., 2013. Hunter–Gatherer Cattle-Keepers of Early Neolithic El Adam Type from Nabta Playa: Latest Discoveries from Site E–06–1. *African Archaeological Review* 30: 253-284.
(54) Marinova, E., Linseele, V. & Vermeersch, P.M., 2008. Holocene environment and subsistence patterns near the Tree Shelter, Red Sea Mountains, Egypt. *Quaternary Research* 70: 392-397.
(55) Gautier, A., 2014. Animal remains from the hidden valley Neolithic site, Farafra Oasis, Egypt. *In*: Barich, B.E., Lucarini, G., Hassan, F.A. & Hamdan, A.M. (Eds.), *From lake to sand, the archaeology of Farafra Oasis, Western Desert, Egypt*. Firenze, All'Insegna del Giglio, pp. 369-374.
(56) Vermeersch, P.M., Linseele, V., Marinova, E., Van Neer, W., Moeyersons, J. & Rethemeyer, J., 2015. Early and Middle Holocene Human Occupation of the Egyptian Eastern Desert: Sodmein Cave. *African Archaeological Review* 32 : 465-503.
(57) Close, A., 2002. Sinai, Sahara, Sahel: The introduction of domestic caprines to Africa. *In* Jennerstrasse 8 (Eds.), *Tides of the Desert-Gezeiten der Wüste*. Henrich-Barth-Institut, Cologne, pp. 459-469.
(58) Marshall, F. & Hildebrand, E. 2002. Cattle Before Crops: The Beginnings of Food Production in Africa. *Journal of World Prehistory* 16: 99-143.
(59) Lesur, J., Hildebrand, E.A., Abawa, G. & Gutherz, X., 2014. The advent of herding in the Horn of Africa: New data from Ethiopia, Djibouti and Somaliland. *Quaternary International* 343: 148-158.

(60) Wendorf, F., 1968. Site 117: A Nubian Final Paleolithic graveyard near Jebel Sahaba, Sudan. *In*: Wendorf, F. (ed), *The Prehistory of Nubia, Volume 2*. Fort Burgwin Research Center and Southern Methodist University Press, Dallas, pp. 954-995.
(61) Solecki, R., de Heinzelin, J., Stigler, R.L., Marks, A.E., Paepe R. & Guichard, J., 1963. Preliminary Statement of the Prehistoric Investigations of the Columbia University Nubian Expedition in Sudan, 1961-1962. *Kush* 11: 70-92.
(62) Wendorf, F., 1968. *The Prehistory of Nubia*. *Volume 1 & 2*. Fort Burgwin Research Center and Southern Methodist University Press, Dallas.
(63) Wendorf, F., Shiner, J.L., Marks, A.E., de Heinzelin, J., Chmielewski, W. & Schild, R., 1966. The 1965 Field Season of the Southern Methodist University. *Kush* 14: 16-24.
(64) Becker, M. & Wendorf, F., 1993. A Microwear Study of a Late Pleistocene Qadan Assemblage from Southern Egypt. *Journal of Field Archaeology* 20 (4): 389-398.
(65) Zazzo, A., 2014. Bone and enamel carbonate diagenesis: A radiocarbon prospective. *Palaeo* 3: 168-178.
(66) Grine, F.E., 2016. The Late Quaternary Hominins of Africa: The Skeletal Evidence from MIS 6-2. *In*: Jones, S.C. & Stewart, B.A. (eds.), *Africa from MIS 6-2: Population Dynamics and Paleoenvironments*. Springer, pp. 323-381.
(67) Lahr, M.M., Rivera, F., Power, R.K., Mounier, A., Copsey, B., Crivellaro, F., Edung, J.E., Maillo Fernandez, J.M., Kiarie, C., Lawrence, J., *et al.* 2016. Inter-group violence among early Holocene hunter-gatherers of West Turkana, Kenya. *Nature* 529: 394-398.
(68) Kissel, M & Kim, NC., 2019. The emergence of human warfare: Current perspectives. *American Journal of Physical Anthropology* 168 (S67): 141-163.
(69) Zazzo, A. & Saliège, J.-F., 2011. Radiocarbon dating of biological apatites: A review. *Palaeogeography, Palaeoclimatology, Palaeoecology* 310: 52-61.
(70) Dal Sasso, G., Zerboni, A., Maritan, L., Angelini, I., Compostella, C., Usai, D. & Artioli, G., 2018. Radiocarbon dating reveals the timing of formation and development of pedogenic calcium carbonate concretions in Central Sudan during the Holocene. *Geochimica et Cosmochimica Acta* 238: 16-35.
(71) Wendorf, F., 1968. Late Paleolithic Sites in Egyptian Nubia. *In*: Wendorf, F. (ed), *The Prehistory of Nubia, Volume 2*. Fort Burgwin Research Center and Southern Methodist University Press, Dallas, pp. 791-953.
(72) Smith, M.J., Brickley, M.B. & Leach, S.L., 2007. Experimental evidence for lithic projectile injuries: improving identification of an under-recognised phenomenon. *Journal of Archaeological Science* 34: 540-553
(73) O'Driscoll, C.A. & Thompson, J.C., 2014. Experimental projectile impact marks on bone: implications for identifying the origins of projectile technology. *Journal of Archaeological Science* 49: 398-413.
(74) Duches, R., Nannini, N., Romandini, M., Boschin, F., Crezzini, J. & Peresani, M., 2016. Identification of Late Epigravettian hunting injuries: Descriptive and 3D analysis of experimental projectile impact marks on bone. *Journal of Archaeological Science* 66: 88-102.
(75) Morel, P., 2000. Impacts de chasse et archéozoologie : quelques observations expérimentales. *In*: Bellier, C., Cattelain, P. & Otte, M. (eds), *La chasse dans la Préhistoire*, ERAUL 51: 54-59.
(76) Pétillon, J.-M. & Letourneux, C., 2003. Au retour de la chasse... *Préhistoires Méditerranéennes* 12: 173-188.

(77) Castel, J.-C., 2008. Identification des impacts de projectiles sur le squelette des grands ongulés. *Annales de Paléontologie* 94: 103-118.
(78) Shipman, P. & Rose, J., 1983. Early Hominid Hunting, Butchering, and Carcass-Processing Behaviors: Approaches to the Fossil Record. *Journal of Anthropological Archaeology* 2: 57-98.
(79) Fernandez-Jalvo, Y. & Andrews, P., 2016. *Atlas of Taphonomic Identifications. 1001+ Images of Fossil and Recent Mammal Bone Modification*. Springer, 359 p.
(80) Potts, R. & Shipman, P., 1981. Cutmarks made by stone tools on bones from Olduvai Gorge, Tanzania. *Nature* 291: 577-580.
(81) Ledermann, S., 1969. *Nouvelles tables-types de mortalités*. Ined, Paris, 260 p.
(82) Bronk Ramsey, C., 2009. Bayesian analysis of radiocarbon dates. *Radiocarbon* 51(1): 337-360.
(83) Reimer, P., Austin, W., Bard, E., Bayliss, A., Blackwell, P., Bronk Ramsey, C., Butzin, M., Cheng, H., Edwards, R., Friedrich, M., *et al.*, 2020. The IntCal20 Northern Hemisphere radiocarbon age calibration curve (0–55 cal kBP). *Radiocarbon* 62, DOI:10.1017/RDC.2020.41.
